# The Effects of Apelin and Elabela Ligands on Apelin Receptor Distinct Signaling Profiles

**DOI:** 10.3389/fphar.2021.630548

**Published:** 2021-03-04

**Authors:** Yunlu Jiang, Maocai Yan, Chunmei Wang, Qinqin Wang, Xiaoyu Chen, Rumin Zhang, Lei Wan, Bingyuan Ji, Bo Dong, Huiyun Wang, Jing Chen

**Affiliations:** ^1^ Neurobiology Key Laboratory of Jining Medical University in Colleges of Shandong, Jining Medical University, Jining, China; ^2^ School of Pharmacy, Jining Medical University, Shandong, China; ^3^ Department of Physiology, Shandong First Medical University, Shandong, China; ^4^ Department of Cardiology, Shandong Provincial Hospital Affiliated to Shandong First Medical University, Jinan, China; ^5^ Division of Biomedical Sciences, Warwick Medical School, University of Warwick, Coventry, United Kingdom

**Keywords:** apelin, Elabela, arrestin, apelin receptor, biased signaling

## Abstract

Apelin and Elabela are endogenous peptide ligands for Apelin receptor (APJ), a widely expressed G protein-coupled receptor. They constitute a spatiotemporal dual ligand system to control APJ signal transduction and function. We investigated the effects of Apelin-13, pGlu_1_-apelin-13, Apelin-17, Apelin-36, Elabela-21 and Elabela-32 peptides on APJ signal transduction. Whether different ligands are biased to different APJ mediated signal transduction pathways was studied. We observed the different changes of G protein dependent and β-arrestin dependent signaling pathways after APJ was activated by six peptide ligands. We demonstrated that stimulation with APJ ligands resulted in dose-dependent increases in both G protein dependent [cyclic AMP (cAMP), Ca^2+^ mobilization, and the early phase extracellular related kinase (ERK) activation] and β-arrestin dependent [GRKs, β-arrestin 1, β-arrestin 2, and β2 subunit of the clathrin adaptor AP2] signaling pathways. However, the ligands exhibited distinct signaling profiles. Elabela-32 showed a >1000-fold bias to the β-statin-dependent signaling pathway. These data provide that Apelin-17 was biased toward β-arrestin dependent signaling. Eabela-21 and pGlu_1_-Apelin-13 exhibited very distinct activities on the G protein dependent pathway. The activity profiles of these ligands could be valuable for the development of drugs with high selectivity for specific APJ downstream signaling pathways.

## Introduction

Apelin receptor (also known as APJ, APLNR, and AGTRL1) is a member of the class A family of G protein-coupled receptor (GPCR), which was first reported by O’Dowd et al. (1993). APJ, which is structurally similar to angiotensin II type l receptor (AT1), is also called putative receptor protein related to AT1 (De Mota et al., 2000). Apelin is an endogenous ligand of APJ that was originally purified from bovine stomach tissue by Tatemoto et al. (1998). The human Apelin gene (APLN) encodes a precursor protein consisting of 77 amino acid residues (Tatemoto et al., 1998), with a signaling peptide at the N-terminus and a number of paired basic amino acids in the central region. This precursor can be hydrolyzed by proteases (for example: angiotensin converting enzyme-2 (ACE2)) (Vickers et al., 2002) into bioactive peptides including Apelin-55, Apelin-36, Apelin-17, and Apelin-13 (Kleinz and Davenport, 2005; O’Carroll et al., 2013). The N-terminal glutamine residue of Apelin-13 is pyroglutamylated, producing the pyroglutamyl form of Apelin-13 ([Pyr1]-apelin-13 (Synonyms: [pGlu1]-Apelin-13) (Lee et al., 2000). The pGlu_1_-apelin-13 has been shown to be the predominant isoform in the human cardiovascular system and human plasma using mass spectrometry to distinguish the isoforms (Read et al., 2019). Shin et al demonstrated that the bioactive Apelin-55 isoform expands the repertoire of therapeutic targets for the apelinergic system (Shin et al., 2017). The Apelin/APJ system is widely expressed in human tissues and regulates a variety of important biological functions, including blood vessel dilation, blood pressure, myocardial contractility, division and proliferation of endothelial cells, angiogenesis, fluid balance, and insulin secretion (Tatemoto et al., 1998; Japp and Newby, 2008; Kidoya and Takakura, 2012; Loot and Fleming, 2013; Wu et al., 2014; Cao et al., 2015; He et al., 2015; Marsault et al., 2019).

Elabela (also known as apela or toddler), which has little sequence homology with Apelin, has recently been identified as the second endogenous APJ ligand. Elabela was first discovered in zebrafish embryos as a factor involved in cardiac development, it has since been shown to have activity in adult mammalian systems (Murza et al., 2016) and its expression is altered in disease (Yang et al., 2017). Elabela is highly expressed during embryogenesis and in adult kidney. Elabela is also derived from a precursor of 54 amino acids. Its 13 C-terminal amino acids are highly conserved among all species, and its 22 N-terminal amino acids may be truncated to form a 32-aa mature peptide (ELA32), a novel peptide hormone (Chng et al., 2013; Pauli et al., 2014; Chaves-Almagro et al., 2015), Beside 32 amino acid isoform (ELA-32), two shorter isoforms consisting of 21 and 11 amino acid corresponding with the C-terminus of ELA-32 have been reported to be functionally active (Chng et al., 2013; Xie et al., 2014). Elabela an endogenous agonist of the APJ in the adult cardiovascular system (Yang et al., 2017). In lower vertebrates, it is critical for endoderm and cardiac development, and it is also expressed in human stem cells and in the prostate and kidney (Chng et al., 2013; Deng et al., 2015; Wang et al., 2015). As an endogenous ligand of APJ, Elabela exerts its biological functions by activating APJ. Elabela can inhibit cyclic AMP (cAMP) production, activate the extracellular signal-regulated kinases (ERK1/2), and mobilize intracellular calcium through APJ internalization (Peverelli et al., 2014). Binding of Elabela to APJ has been shown to increase cardiac contractility in an ERK1/2-dependent manner (Perjés et al., 2016).

Apelin and Elabela competitively act on APJ to fine-tune the modulation of certain physiological activities. Signal transduction upon receptor activation depends on the binding ligand and the structure of the receptor. Upon the binding of different ligands, APJ may transduce a signal either through G protein dependent pathways or through β-arrestin dependent pathways (Azzi et al., 2003; Wei et al., 2003). Ligands that lead to biased activation of a receptor are called biased ligands (Rajagopal et al., 2010). Murza et al. demonstrated that some macrocyclic Apelin ligands are biased toward APJ receptor agonists (Murza et al., 2017) and Elabela also that binds to APJ, activates the Gαi1 and β-arrestin2 signaling pathways, and induces receptor internalization similarly to its parent endogenous peptide (Murza et al., 2016). Elabela plays crucial roles in heart development and disease conditions presumably at time points or at areas of the heart different from Apelin (Kuba et al., 2019). Elabela but not Apelin knockout pregnant mice exhibit preeclampsia-like symptoms, including proteinuria and elevated blood pressure (Sato et al., 2017).

Based on the fact that two different groups of peptides act on a functional APJ, it is presumed that different signal transduction pathways, even biased signal transduction pathways, are generated. The complex properties of biased signaling enrich the function of GPCRs, potentially leading to improved therapeutic properties and reduced side effects or toxicity (DeWire and Violin, 2011; Smith et al., 2018).

Quantitative analysis of the peptide functions of multiple intracellular signaling pathways is a necessary condition for the study of biased agonist (peptide). The APJ preferentially couples to Gi/o and Gq proteins (Bai et al., 2014b), and therefore Apelin and Elabela activation stimulates the canonical signal transduction pathway. In this study, Apelin-13, pGlu_1_-apelin-13, Apelin-17, Apelin-36, Elabela-21 and Elabela-32 peptides were assessed for the G protein dependent (cAMP accumulation, the early phase phosphorylate ERK1/2 and intracellular calcium concentrations) and β-arrestin dependent (GRKs, β-arrestin 1, β-arrestin 2, and β2 subunit of the clathrin adaptor AP2) signaling pathways. To investigate whether different APJ ligands have biases for different APJ-mediated signal transduction pathways. Our research will help us to understand the complexity of apelinergic system and the functional differences between Apelin and Elabela.

## Experimental Procedures

### Materials

#### Peptides

Human Apelin-13, pGlu_1_-apelin-13, Apelin-17, Apelin-36, Elabela-21, and Elabela-32 peptides were obtained from Phoenix Pharmaceuticals (Belmont, CA, United States). [^125^I]-apelin-13 was purchased from Perkin Elmer (Wellesley, MA, United States).

#### Reagents

Geneticin (G418) and Lipofectamine 2000 were purchased from Invitrogen/Thermo Fisher Scientific (Waltham, MA, United States). Antibodies: The β_1/2_-arrestin antibodies, hemagglutinin (HA) antibody, Anti-p-ERK antibody and Anti-ERK antibody were obtained from Cell Signaling Technology (Danvers, MA, United States). The rabbit Anti-APJ Receptor antibody was obtained Abcam (Cambridge, United Kingdom). The β-actin antibody and secondary antibodies were purchased from ZSGB Bio (Beijing, China).

#### Plasmids

The pcDNA3.1 (+) APJ plasmid was obtained from Missouri S&T cDNA Resource Center (Rolla, MO, United States). APJ-Venus, APJ-Renilla luciferase (Rluc), β-arrestin1-Rluc, and β-arrestin2-Rluc expression plasmids were constructed as described (Chen et al., 2014). Plasmids GRK2-GFP2 and GRK5-GFP2 were kindly provided by Prof. Christian E. Elling, 7TM Pharma A/S, 2970 Horsholm, Denmark. A mammalian expression vector that includes CAMYEL (cAMP sensor using YFP-Epac-RLuc) was obtained from ATCC (LGC Standards, Middlesex, United Kingdom). The β_2_-adaptin EYFP (AP2, β2 subunit of the clathrin adaptor) was kindly provided by Dr. Michel Bouvier (Université de Montréal, Canada).

#### Cell Culture and Transfection

Human embryonic kidney (HEK) 293 cells were routinely cultured in Dulbecco’s modified Eagle’s medium (DMEM) containing 10% fetal bovine serum (FBS). These cultures were maintained at 37°C in a 5% CO_2_ incubator. For stable transfection, cells were transfected with recombinant plasmids using Lipofectamine 2000 reagent following the manufacturer’s instructions (Li et al., 2012; Bai et al., 2014a; Chen et al., 2015), and then cultured in selective media supplemented with G418 (0.5 mg/ml) for 7–9 weeks (Liu et al., 2016; Ji et al., 2017). Western blotting was performed on the stable cell lines to ensure the expression of APJ. The stable transfected cell line is the application of cAMP, intracellular calcium and ERK1/2 activation assay.

### Ligand Affinity Experiments

#### Radioligand Binding and Competition Assays

##### APJ Membrane Preparation

APJ membrane preparations were obtained from transiently transfected HEK293 cells. HEK293 cells were cultured in complete DMEM (DMEM plus 10% FBS) for 24 h, until they reached at least 70% confluence. Transfection reagent containing 20 μg of the APJ expression plasmid was added, and the cells were incubated for another 24 h. The cells were then harvested into 25 ml homogenization buffer (HB) [250 mM sucrose and 25 mM HEPES (pH 7.3); adjusted to pH 7.5 at 4°C and then filtered] supplemented with 250 μL phenylmethylsulfonyl fluoride (PMSF), homogenized using an IKA T25 Ultra Turrax (24,000 rpm), and centrifuged at 6,025 rpm for 15 min at 4°C. The supernatant was collected carefully, and the pellet was suspended in HB and centrifuged again. The resulting APJ membrane preparation was aliquotted at 2 mg/vial with storage buffer (50 mM Tris-HCL (pH 7.4), 0.5 mM EDTA, 10 mM MgCl2, 10% sucrose) and stored at −80°C.

##### Membrane Titration

The HEK-293/APJ membrane preparation (40, 20, 10, or 5 μg/well in a volume of 120 µL) was incubated with 15 μL [^125^I]-apelin-13 (0.1 nM) with or without 15 μL cold Apelin-13 (100 nM final) in binding assay buffer [50 mM HEPES (pH 7.4), 5 mM MgCl_2_, 1 mM CaCl_2_, 0.2% bovine serum albumin (BSA); filtered and stored at 4°C] at 30°C for 2 h. The binding reaction was stopped by rapid filtration through polyethyleneimine (PEI)-coated GF/B plates using a cell harvester, and the plates were washed three times with ice-cold wash buffer [50 mM HEPES (pH 7.4), 500 mM NaCl, and 0.1% BSA; filtered and stored at 4°C]. The filtration plates were dried at 37°C for 2 h; 50 μL scintillation cocktail (Perkin Elmer, Wellesley, MA, United States), was added to each well; and the radioactivity was measured using a Perkin Elmer 1450 MicroBeta TriLux Microplate Scintillation and Luminescence Counter.

##### Saturation Binding Assay

HEK-293/APJ membrane preparation (20 μg/well in a 120 µL volume) was incubated with 15 μL [^125^I]-apelin-13 (2-fold dilutions starting at 1 nM) with or without 15 μL cold Apelin-13 (100 nM final) in binding assay buffer at 30°C for 2 h. The binding reaction was stopped, the plates were washed and dried, the scintillation cocktail was added, and the radioactivity was measured according to the method described above (Supplementary Figure S1A).

##### Competition Binding Assay

HEK-293/APJ membrane preparation (20 μg/well in a 120 µL volume) was incubated with 15 μL [^125^I]- apelin-13 (0.1 nM) with or without 15 μL test peptides (5-fold dilutions starting at 10 µM) in binding assay buffer at 30°C for 2 h. The binding reaction was stopped, the plates were washed and dried, the scintillation cocktail was added, and the radioactivity was measured as described above. The *K*_i_ values were calculated from the corresponding 50% inhibitory concentrations (IC_50_) according the following formula:

$$K_{i}={\text{IC}}_{50}/\left[1+\left(\left[L\right]/K_{d}\right)\right]={\text{IC}}_{50}/\left(1+0.1/0.5265\right)={\text{IC}}_{50}/1.12$$

Where in *K*_*d*_ was obtained from the saturation binding assay (Supplementary Figure S1A).

#### Bioluminescence Resonance Energy Transfer Measurements

To detect the potencies of different peptide ligands for APJ in living cells, real-time kinetic bioluminescence resonance energy transfer (BRET) assays were carried out as follows. HEK293 cells were grown to 80–90% confluence in 24-well plates and transiently transfected with various Rluc-, EYFP-, or Venus-tagged constructs (Chen et al., 2014; Liu et al., 2016). Approximately 36 h after transfection, the long-acting luciferase substrate EnduRen (E6481, Promega, Beijing Biotech Co., Ltd.) was added to each well at a final concentration of 60 μM and cells were incubated for 2 h at 37°C in a 5% CO_2_ incubator. BRET experiments were carried out using a Mithras LB941 plate reader (Berthold Technologies, Bad Wildbad, Germany) with three filters: a Rluc filter (400–475 nm), a Venus filter (515–575 nm) and an EGFP filter (500–550 nm) (Liu et al., 2016; Ji et al., 2017). The BRET ratio was calculated as the ratio of the emission at 535 ± 30 nm to that at 485 ± 20 nm. The results are expressed as the mBRET (mBRET = 1,000 × BRET ratio) (Pfleger et al., 2006a; Pfleger et al., 2006b). We use high glucose as a negative control group.

#### BRET-Based cAMP Biosensor Method Assay for cAMP

**FIGURE 1:**
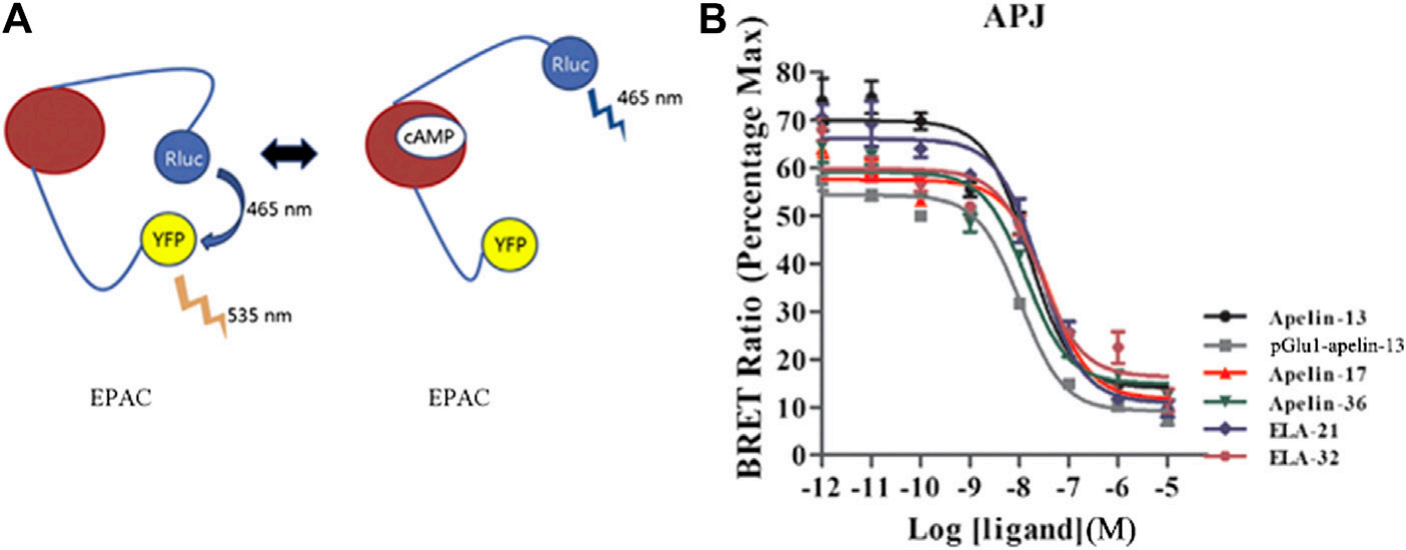
Intracellular cAMP production induced by the six agonists. **(A)** A schematic diagram of YFP-cAMP-Rluc fusion expression vector. **(B)** In HEK293 cells stably expressing APJ, the cAMP dose-response curve was measured at 20 min after stimulation with six peptide. *p* < 0.05 was considered as statistically significant. Data represent mean ± SEM from four independent experiments (Supplementary Table S1).

The cAMP concentration was measured using a BRET-based cAMP biosensor (Barak et al., 2008; Ji et al., 2017) in HEK293 cells transiently co-expressing YFP-Epac-RLuc and APJ. The sensor is composed of an N-terminal truncated variant of EPAC tagged with Rluc and YFP at N-terminal and C-terminal, respectively. The sensor, designated “CAMYEL” (cAMP sensor using YFP-Epac-RLuc), changes conformation in response to increasing levels of cAMP, resulting in a loss of BRET intensity (Figure 1). At 24 h after transfection, cells were digested and plated into a 96-well microplate for 24 h following adding the substrate coelenterazine H into the medium at the final concentration of 5 µM and the nonspecific phosphodiesterase inhibitor IBMX (40 μM) for 20 min, and the cells were stimulated for 10 min with forskolin (10 μM). Cells were then stimulated with containing Apelin-13, pGlu_1_-apelin-13, Apelin-17, Apelin-36, Elabela-21 and Elabela-32 peptides (0.01–10,000 nM) for 20 min, BRET readings were collected on a Mithras LB940 plate reader (Berthold Technologies, Bad Wildbad, Germany).

#### Intracellular Calcium Assay

HEK293 cells stably expressing APJ were plated at 10^4^ cells per well in 96-well black microplates pretreated with a 0.1 mg/ml solution of poly-d-lysine for 24 h. Calcium fluorescence signals in cells were measured using the Fluo-4 NW calcium assay kit (Thermo Fisher Scientific) according to the manufacturer’s instructions (Chen et al., 2014; Wang et al., 2017). Before detection, cells were washed twice with assay buffer (1 × HBSS, 20 mM HEPES). One hundred microlitres of the dye loading solution was added to each well of a 96-well plate. Cells were incubated at 37°C in 5% CO2 humidified atmosphere for 30 min, then at room temperature for an additional 30 min. Cells were stimulated with six ligands (100 nM) for 5 min, then calcium signals were immediately measured on a Mithras LB940 plate reader (Berthold Technologies, Bad Wildbad, Germany) with an excitation wavelength of 485 nm and an emission wavelength of 525 nm. Dose response curve: cells were stimulated with different concentrations of six peptides for 30 s, and calcium fluorescence was detected. The calculation of the calcium fluorescence ratio was described previously (Chen et al., 2014).

#### Real Time Kinetic and Dose-Response BRET Determination

HEK293 cells were transiently co-transfected with the designated Rluc and GFP2 or Venus or EYFP-labeled constructs. Twenty-four hours after transfection, the cells were harvested in phenol red free medium containing 5% FCS in HEPES buffer and seeded in 96 well white microplate coated with poly-d-lysine. After 24 h, cells were washed twice in PBS and re-suspended in PBS containing 0.5 mM MgCl2 and 0.1% glucose. Coelenterazine h substrate was added at a final concentration of 5 μM in the total volume of 50 μL/well. Kinetic-response and dose-response curve of the recruitment of GRKs or β-arrestin 1 and β-arrestin 2 or AP2 to the APJ by six peptides measured by BRET. HEK293 cell transiently transfected 1) APJ Rluc-tagged and GRK2-GFP2 or GRK5-GFP2 2) APJ Venus-tagged and β-arrestin1-Rluc or β-arrestin2-Rluc 3) APJ Rluc-tagged and β2-adaptin EYFP-tagged (AP2) were treated with increasing doses of six peptide. The BRET signals between receptor and GRKs/β-arrestin1 and 2/AP2 were recorded at 10 min (GRK2/5) or 15 min (β-arrestin1 and) or 30 min (AP2) after agonist stimulation. We use high glucose as a control group. BRET assays were performed as described previously (Xue et al., 2018). The observed BRET ratios between interacting proteins were normalized by subtracting the background BRET ratios; the samples treated with vectors represented the background ratio. This signal is defined as the ligand induced BRET ratio.

#### ERK1/2 Activation Assay

HEK293 cells expressing APJ were spilt into 6-well plates at a density of 10^5^ cells per well and cultured until reaching approximately 90% confluence. The cells were treated with different concentrations of peptide ligands (0.01–1,000 nM). The cells were washed twice with Phosphate-buffered saline (PBS) and lyzed in RIPA lysis buffer (50 mm Tris–HCl, pH 7.41, 50 mM NaCl, 1 mM EDTA, 10 mM MgCl2, 1 mM DTT, 1 mM PMSF, 1 μg/ml leucine, 2 μg), 1.4 μg/ml gastrin A and 0.5% (v/v) NP-40). After centrifugation at 14,000 rpm at 4°C for 30 min, the supernatant was collected and separated by 10% SDS-PAGE. Gel transfer takes about 60 min at 250 mA. The nitrocellulose membrane was sealed with 5% skim milk dissolved in 0.1% Triton X-100 of PBS. The primary ERK1/2 antibody was diluted with 5% bovine serum albumin (BSA) at 1:2,000, and the second antibody was diluted with 1:5,000. The ECL reagent was applied to the membrane. The films were scanned by Vanoscan Lide 700F scanner and analyzed by scion image software. The dose-response curve was fitted using the data detected at 5 min after stimulation. PcDNA3.1 (+) vector was used as negative control group to exclude false positive results (Supplementary Figure S1B).

### Analysis of Ligand Bias

The concentration-response data for the ligands in various assays were analyzed to determine the biases of the ligands for G protein dependent signaling or β-arrestin dependent signaling, according to a previously published method (Kenakin et al., 2012; Hager et al., 2016; Hothersall et al., 2017). Briefly, the concentration-response data for each ligand were fitted to the Black–Leff operation model to obtain the values of *τ* and *K*_A_, using the following equation:

$$\text{Response}=\frac{{\left[A\right]}^{n}\tau^{n}E_{m}}{{\left[A\right]}^{n}\tau^{n}+(\left[A\right]K_{\text{A}}{)}^{n}}$$

where [A] is the concentration of the agonist, *E*_m_ is the maximal response of the system, *n* is the transducer slope, and *K*_A_ and *τ* describe the affinity and efficacy of the agonist, respectively. In the data fitting, the reparametrized form of the equation proposed by Kenakin et al. (2012) was used:

$$\text{Response}=\frac{E_{m}}{1+\exp\left\{n\ln\left[1+\exp\left(\beta_{1}\right)/\left[A\right]\right]-n\beta_{2}\right\}}$$

where *β*_1_ and *β*_2_ are the natural logarithm of *K*_A_ and *τ*, respectively, and *E*_m_ was set to 1. Thus, for each concentration-response curve, a non-linear regression was performed to fit the above equation to give *β*_1_ and *β*_2_ (as well as *n*). Log (*τ*/*K*_A_) values (“transduction coefficients”) for each ligand vs. each pathway were obtained. The endogenous APJ ligand Apelin-13 was chosen as the reference ligand, and intracellular calcium release was selected as the reference pathway. Then, the difference in the transduction coefficient of each ligand relative to Apelin-13 [Δlog (*τ*/*K*_A_)] was calculated. The ΔΔlog (*τ*/*K*_A_), or Log (Bias), which is the logarithm of the bias factor for a given pathway over a reference pathway (here, intracellular calcium release), was calculated for each ligand as Δlog (*τ*/*K*_A_)–Δlog (*τ*/*K*_A_)_Calcium_. The 95% confidence interval (CI) was expressed as the mean ± standard error; Log (Bias) values, whose 95% CI crosses 0, were considered statistically insignificant.

### Statistical Analysis

The data were analyzed using GraphPad Prism v5.0. All the data are presented as the mean ± SEM. Differences between groups were compared by one-way analysis of variants (ANOVA), followed by post-hoc tests (Tukey’s test), When *p* < 0.05, the difference was considered significant. All experiments were repeated at least four times. The dose-response curves were generated using non-linear fitting model log (agonist) vs. response (three-parameters), with Hill Slope held to 1, i.e., Y = Bottom + (Top-Bottom)/(1 + 10^(LogEC50-X)).

## Results

### Ligand Binding Affinity

**FIGURE 2:**
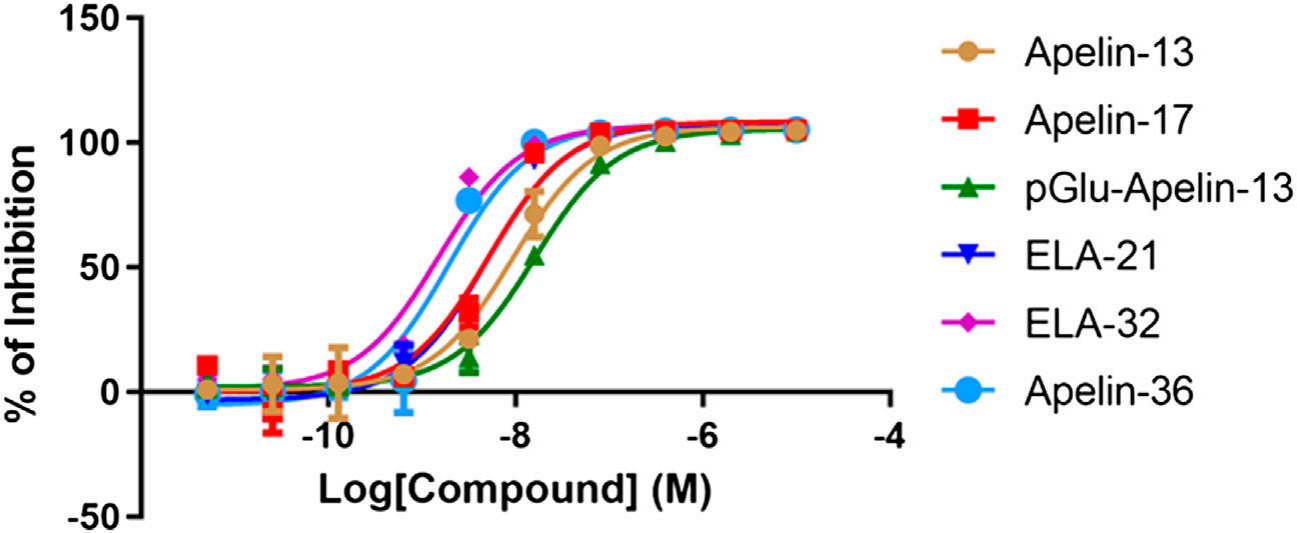
Radio-ligand competition results of the six peptides with [^125^I]-apelin-13. All data points were in triplicates and the standard errors were displayed in error bars. The inhibition rates have been normalized to Apelin-13.

The radioligand competition assay results are shown in Figure 2 and Table 1. Because of the lack of suitable Elabela radioactive ligand ([^125^I]-apelin-13 radioligand was used in this assay), we indirectly compared the binding patterns of Apelin and Elabela fragments (the six peptide ligands). The binding affinities of all six peptide ligands were comparable, and their inhibition constants (*K*_i_) were in the nM range. Overall, Apelin-36 (1.735 nM) and Elabela-32 (1.343 nM) had the most similar binding affinities; the truncated Apelin peptides [Apelin-13 (8.336 nM), Apelin-17 (4.651 nM), and pGlu_1_-apelin-13 (14.366 nM)] displayed slightly weaker binding affinities than Apelin-36, and the truncated Elabela peptide Elabela-21 (4.364 nM) displayed slightly weaker binding affinity than Elabela-32. Compared with Apelin-13, the structurally modified pGlu_1_-apelin-13 peptide showed further decreased binding ability. Nevertheless, the maximum difference among the *K*_i_ values was approximately 10-fold (Elabela-32 vs. pGlu_1_-apelin-13).

**TABLE 1:** Inhibition constant (Ki) of the six peptide ligands.

	Apelin-13	Apelin-17	pGlu_1_-Apelin-13	ELA-21	ELA-32	Apelin-36
IC_50_ (nM)	9.33 ± 0.09	5.20 ± 0.08	16.0 ± 0.09	4.88 ± 0.07	1.50 ± 0.09	1.94 ± 0.09
*K*_i_ (nM)	8.33 ± 0.03	4.65 ± 0.03	14.36 ± 0.04	4.36 ± 0.02	1.34 ± 0.03	1.73 ± 0.02
p*K*_i_	8.08	8.33	7.84	8.36	8.87	8.76

### cAMP Production

To determine the effects of various ligands on APJ-induced intracellular cAMP production, six ligands (Apelin-13, pGlu_1_-apelin-13, Apelin-17, Apelin-36, Elabela-21, and Elabela-32) were investigated for their effects on cAMP production. After stimulation at 20 min, all six ligands inhibited FSK-induced intracellular cAMP production in a dose-dependent manner, with log IC_50_ values of −7.817 ± 0.363, −7.978 ± 0.409, −7.419 ± 0.341, −7.865 ± 0.346, −7.589 ± 0.352, and −7.59 ± 0.474, respectively. Apelin-17 exhibited significantly higher inhibitory effects than the other ligands, whereas pGlu_1_-apelin-13 was the weakest agonist of this signaling pathway (Figure 1 and Supplementary Figure S2). Therefore, all six ligands could inhibit cAMP production in HEK293 cells by activating APJ; nevertheless, their inhibitory effects differed in strength. The IC_50_ or EC_50_ values of all six ligands on different pathways were summarized in Table 2.

**TABLE 2:** Efficacy or inhibitory potency of each ligand on different pathways.

Ligand	Apelin-13	pGlu_1_-apelin-13	Apelin-17	Apelin-36	Elabela-21	Elabela-32
cAMP production^a^	−7.817 ± 0.363	−7.978 ± 0.409	−7.419 ± 0.341	−7.865 ± 0.346	−7.589 ± 0.352	−7.59 ± 0.474
Calcium release^b^	−8.741 ± 0.1315	−8.846 ± 0.1188	−8.239 ± 0.1468	−8.750 ± 0.1525	−7.960 ± 0.1371	−8.124 ± 0.1285
ERK1/2 phosphorylation^b^	−9.740 ± 0.1943	−8.554 ± 0.2554	−9.124 ± 0.2400	−7.806 ± 0.2022	−8.145 ± 0.1318	−8.570 ± 0.2092
GRK2^b^	−6.899 ± 0.2155	−7.972 ± 0.3283	−8.189 ± 0.2182	−8.867 ± 0.2206	−7.473 ± 0.1371	−8.989 ± 0.2404
GRK5^b^	−6.828 ± 0.2606	6.814 ± 0.3200	−8.579 ± 0.2991	−6.824 ± 0.2365	−7.233 ± 0.2301	−8.114 ± 0.2153
β-arrestin 1^b^	−6.369 ± 0.086	−6.899 ± 0.106	−7.901 ± 0.144	−7.027 ± 0.087	−7.183 ± 0.061	−7.66 ± 0.114
β-arrestin 2^b^	−6.813 ± 0.091	−6.72 ± 0.218	−8.333 ± 0.157	−6.708 ± 0.248	−7.175 ± 0.14	−7.878 ± 0.284
β-arrestin 1/AP2^b^	−7.612 ± 0.1867	−8.091 ± 0.2581	−8.483 ± 0.2184	−7.975 ± 0.2180	−8.043 ± 0.1642	−8.063 ± 0.2934
β-arrestin 2/AP2^b^	−8.379 ± 0.2436	−8.003 ± 0.4018	−7.169 ± 0.3522	−8.961 ± 0.4233	−7.764± 0.3415	−8.866 ± 0.3241

^a^ LogIC_50_ values.

^b^ LogEC_50_ values.

### Intracellular Calcium Release

**FIGURE 3:**
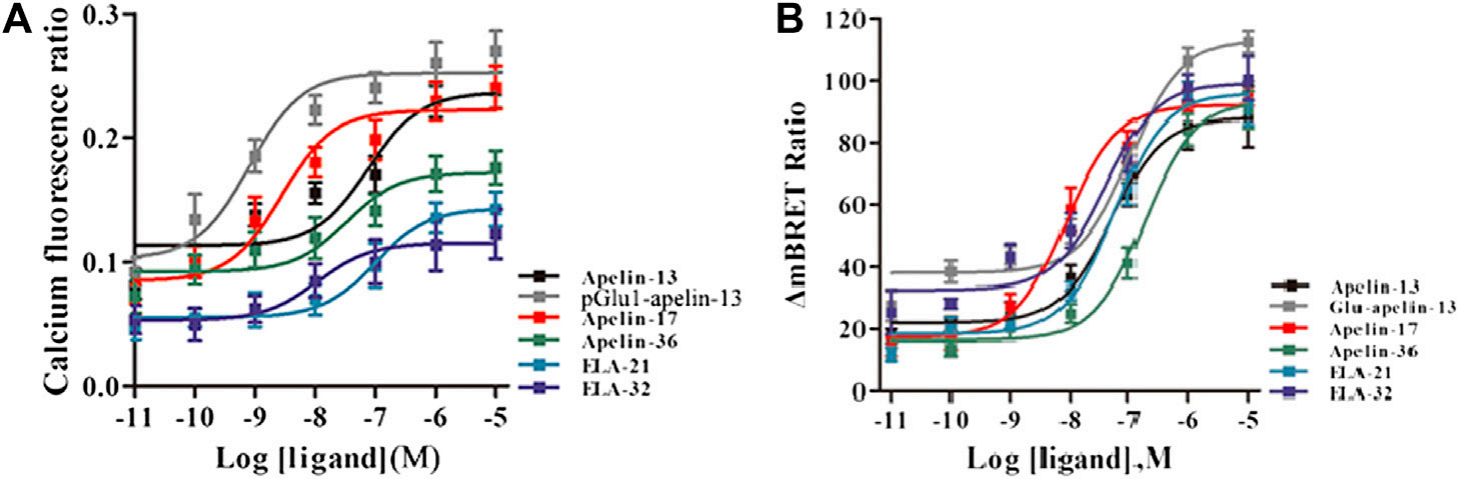
**(A)** The intracellular Calcium production induced by the six agonists. HEK293 cells stably expressed APJ, and in 30 s, the dose response curve stimulated with six peptides was measured. Data represent mean ± SEM from four independent experiments (Supplementary Table S2). With six peptides **(B)** stimulation, the dose-response curve of GRK2 recruited to APJ was measured by BRET (at 15 min). *p* < 0.05 was considered as statistically significant. Data represent mean ± SEM from four independent experiments (Supplementary Table S3).

Intracellular calcium release induced by APJ upon activation by the six ligands was next investigated. All six ligands promoted intracellular calcium release in a dose-dependent manner; pGlu_1_-apelin-13 significantly increased calcium release, while Elabela-32 displayed relatively weak activity (Figure 3A and Supplementary Figure S3). Hence, all six ligands promoted intracellular calcium release via APJ, but to different extents.

### ERK1/2 Phosphorylation

**FIGURE 4:**
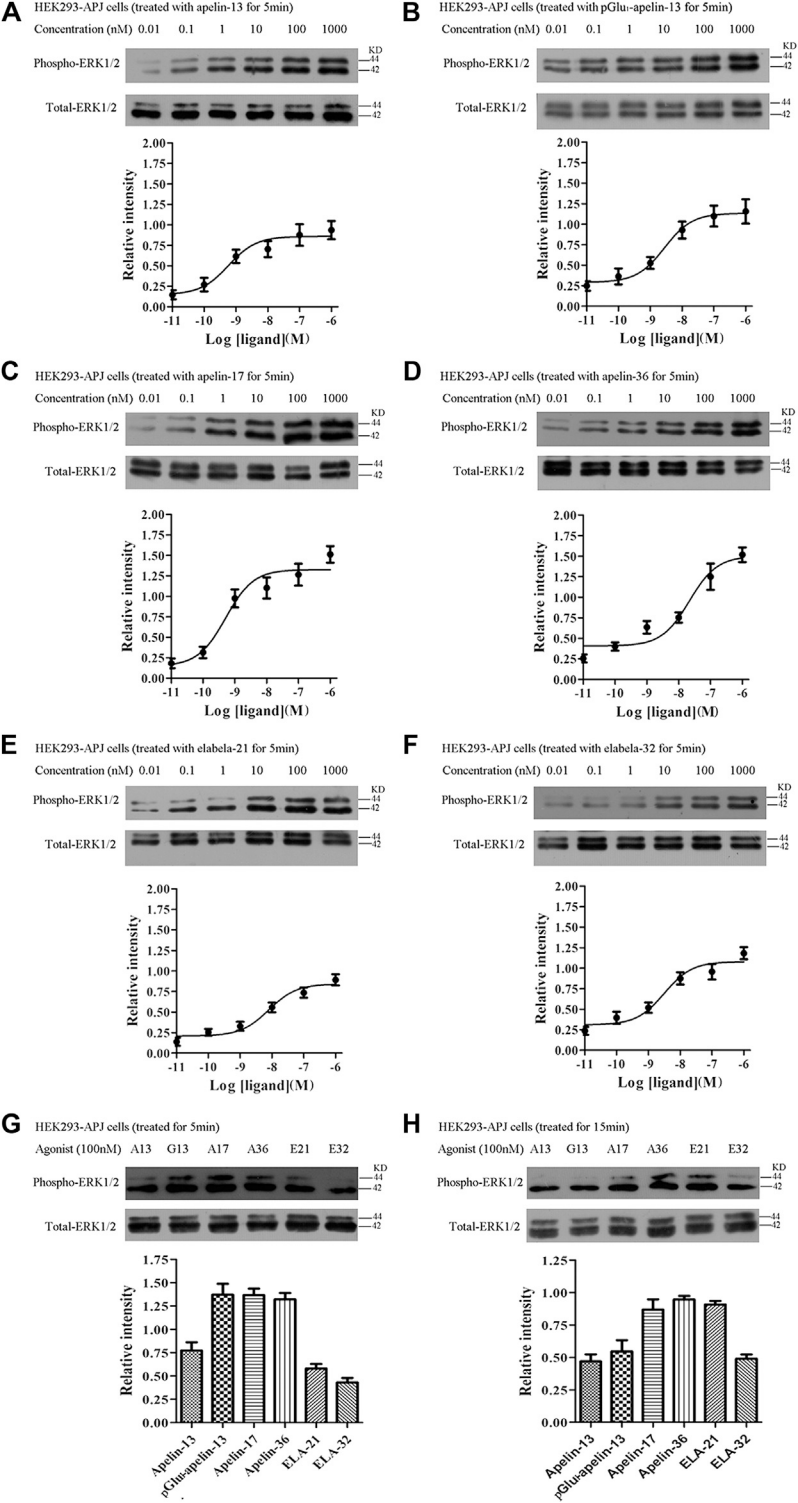
Changes of ERK1/2 phosphorylation in HEK293 cells with stable expression of APJ: **(A–F)** after 5-min stimulation by the six agonists in different concentrations (0.01–1,000 nM) **(G** and **H)** ERK was measured after 5 or 15 min stimulation with six peptides at a concentration of 100 nM. Levels of p-ERK, total ERK were determined by Western blot. The data represent means ± SEM of four independent experiments.

To evaluate ERK1/2 phosphorylation, HEK293 cells stably expressing APJ were stimulated with the six ligands at different concentrations (0.1–1,000 nM), and proteins were extracted for Western blot analysis. HEK293 cells expressing APJ exhibited dose-dependent ERK1/2 phosphorylation (Figures 4A–F) after 5 min of treatment with the six APJ ligands. At a ligand concentration of 100 nM, pGlu_1_-apelin-13, Apelin-17, and Apelin-36 induced significantly higher levels of ERK1/2 phosphorylation than the other ligands at 5 min; however, at 15 min, ERK1/2 phosphorylation in response to Apelin-17, Apelin-36, and Elabela 21 was significantly higher than the activation of ERK1/2 by the other ligands (Figures 4G–H). Therefore, different peptide ligands of APJ result in different kinetics of ERK1/2 phosphorylation.

### The Real-Time, Dynamic Determination of Interactions of APJ With GRK2/5 by BRET

The six peptide ligands (100 nM) were used to stimulate HEK293 cells co-transfected with APJ-Rluc and GRK2-GFP2, and interactions between APJ and GRK2 were determined by BRET. All six ligands induced the APJ–GRK2 interaction dose-dependently, and the signal peaked after approximately 10 min of treatment with the ligands (Supplementary Figures S4A–F). BRET was used to detect six peptide dose-response curves of APJ recruited by GRK2 at 10 min after stimulation. The results showed that the six peptides caused a dose-dependent increase in BRET signal of APJ, indicating that GRK2 was recruited to the activated receptor. The signal induced by pGlu_1_-apelin-13 was significantly higher than that induced by the other ligands (Figure 3B).

**FIGURE 5:**
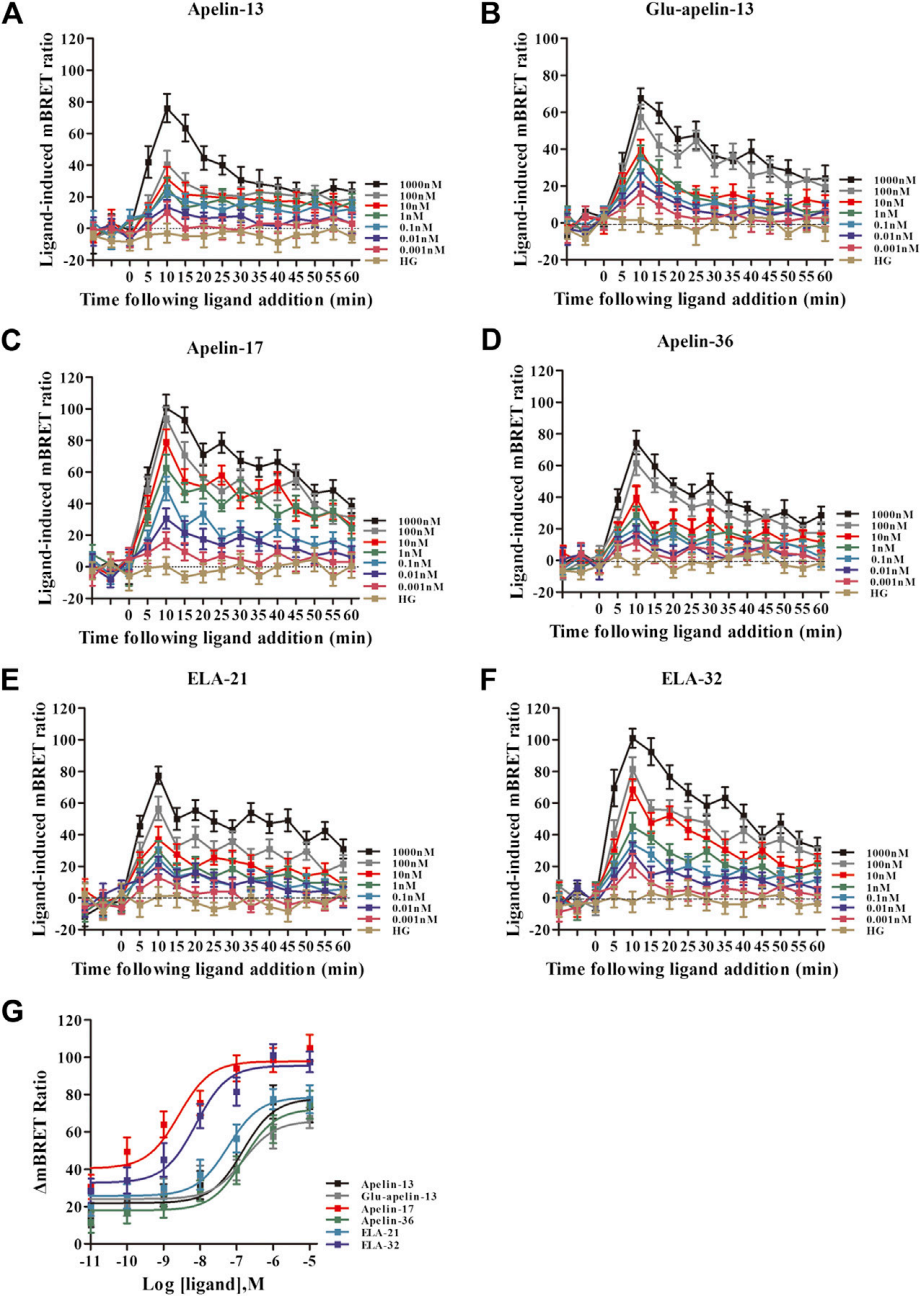
The time-response curve (**(A–F)** for GRK5) and dose-response curve (**(G)** for GRK5) of interactions between APJ and GRK5 induced by the six agonists of different concentrations, obtained from real-time dynamic BRET determination. *p* < 0.05 was considered as statistically significant. Control group (HG, Cells were stimulated with high glucose). With six peptides **(G)** stimulation, the dose-response curve of GRK5 recruited to APJ was measured by BRET (at 15 min). Data represent mean ± SEM from four independent experiments. Statistical analysis (Supplementary Table S4).

Using the same method, interactions of APJ with GRK5 were investigated. Similarly, the BRET signals increased dose-dependently upon treatment with all six peptide ligands and peaked after 10 min of treatment (Figures 5A–F). Dose-response curve of the recruitment of GRK5 to APJ measured showed that six peptide caused a robust dose-dependent. Among the six peptide ligands, Apelin-17 induced a significantly higher BRET signal than the other ligands (Figure 5G).

### Determination of Interactions of APJ With β-arrestin 1 and 2 by BRET

**FIGURE 6:**
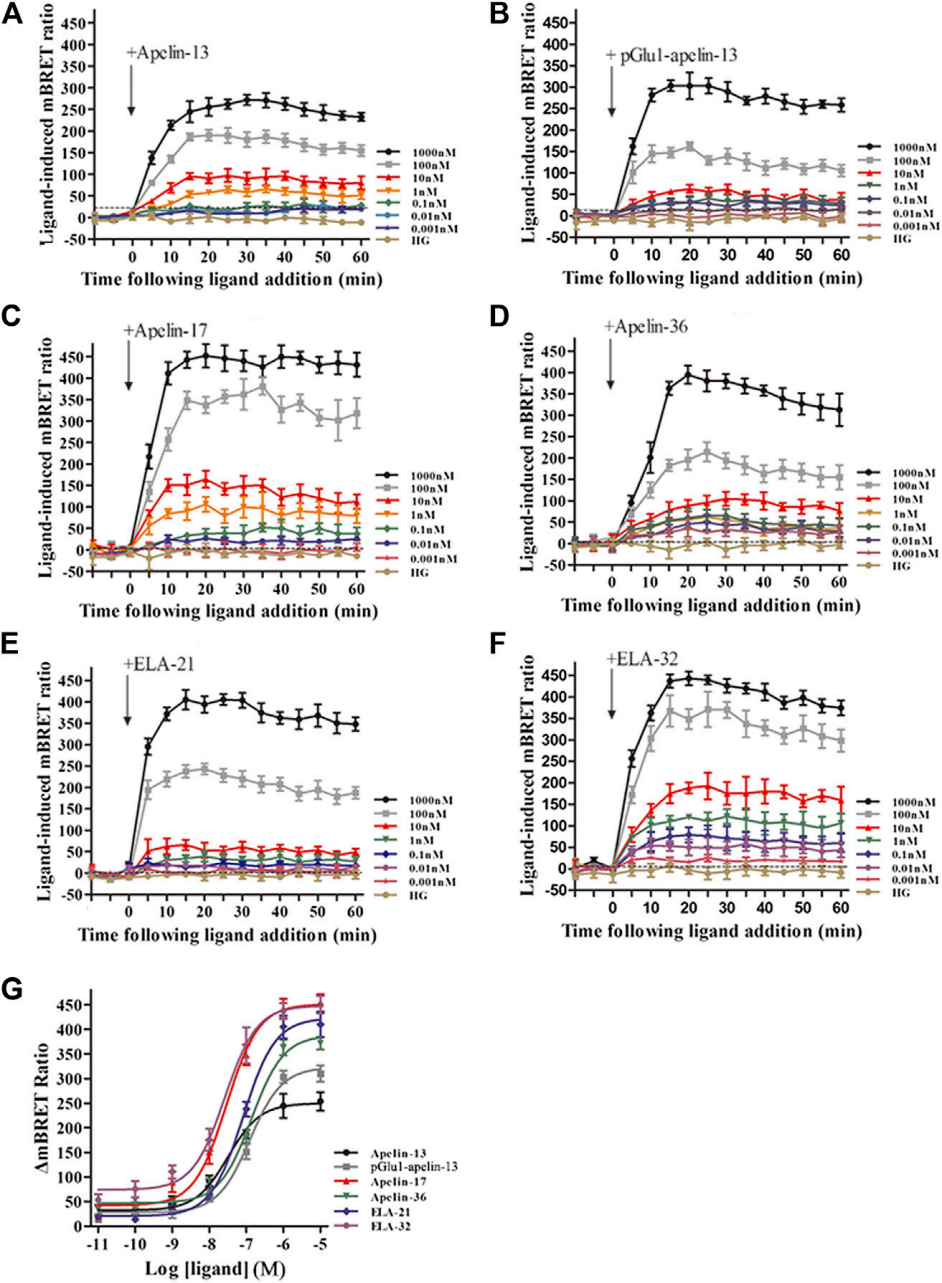
The time-response curve **(A–F)** and dose-response curve **(G)** of interactions between APJ and β-arrestin1 induced by the six agonists in different concentrations, obtained from real-time dynamic BRET determination. *p* < 0.05 was considered as statistically significant. Control group (HG, Cells were stimulated with high glucose). In HEK293 cells expressing APJ and β-arrestin 1, the dose-response curves of the six peptides at 20 min were observed. The data represent means ± SEM of four independent experiments. Statistical analysis (Supplementary Table S5).

BRET technology was employed to determine the effects of the six peptide ligands on HEK293 cells co-transfected with APJ-Venus and β-arrestin 1-Rluc. For all six peptide ligands, the interaction of APJ with β-arrestin 1 increased dose-response dependently and peaked after approximately 15 min of treatment (Figures 6A–F). Similarly, six peptide dose response curves of APJ were detected by BRET at 15 min. The data showed that the six peptides increased the BRET signal of APJ in a dose-dependent manner. The BRET signals induced by Elabela 32 and Apelin-17 were significantly higher than those induced by the other peptide ligands (Figure 6G). The logEC_50_ values of the six agonists (Apelin-13, pGlu_1_-apelin13, Apelin-17, Apelin-36, Elabela-21, and Elabela-32) were −6.369 ± 0.086, −6.899 ± 0.106, −7.901 ± 0.144, −7.027 ± 0.087, −7.183 ± 0.061, and −7.66 ± 0.114, respectively.

**FIGURE 7:**
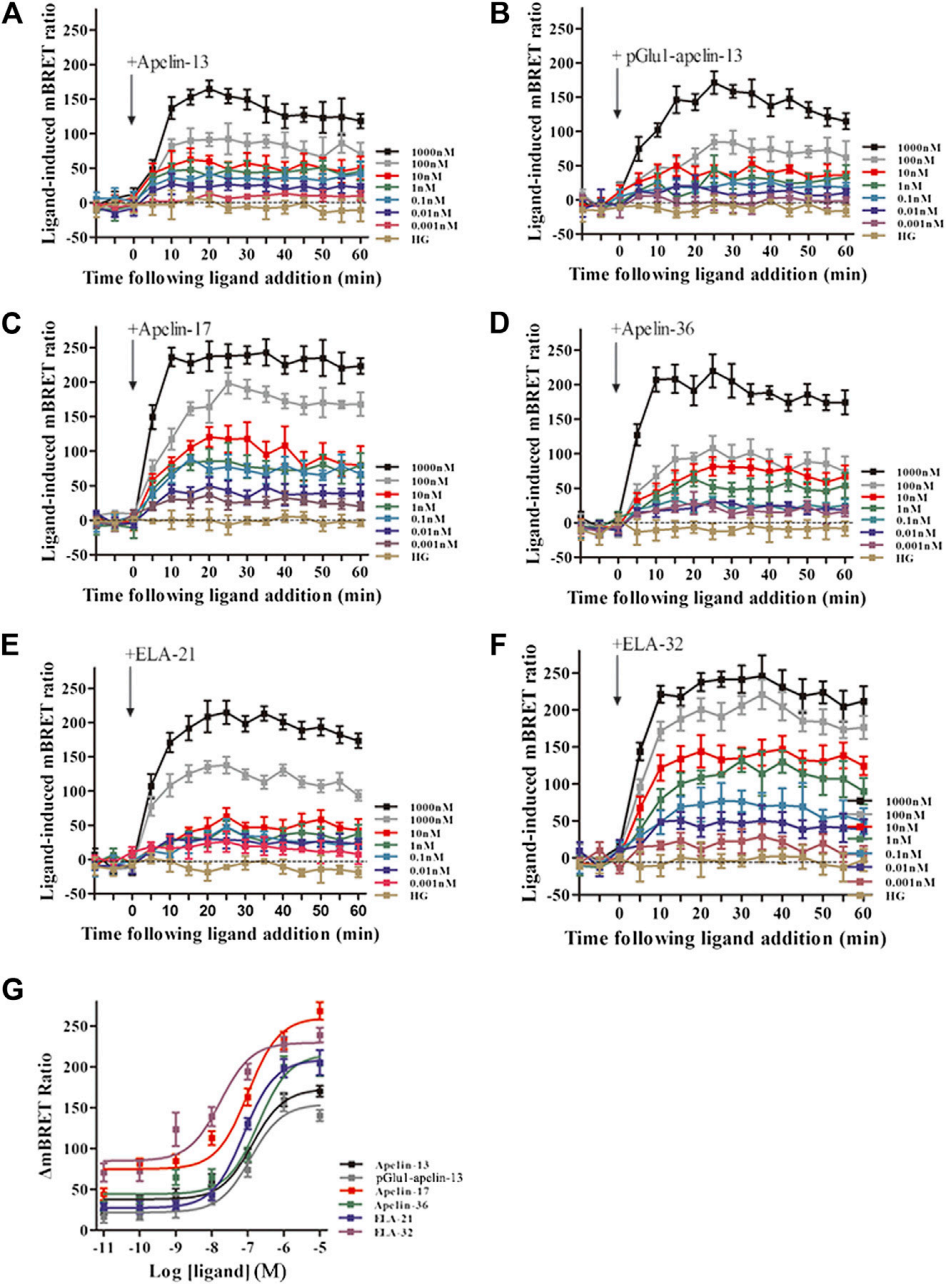
The time-response curve **(A–F)** and dose-response curve **(G)** of interactions between APJ and β-arrestin2 induced by the six agonists in different concentrations, obtained from real-time dynamic BRET determinations. *p* < 0.05 was considered as statistically significant. Control group (HG, Cells were stimulated with high glucose). In HEK293 cells expressing APJ and β-arrestin 2, the dose-response curves of the six peptides at 20 min were observed. The data represent means ± SEM of four independent experiments. Statistical analysis (Supplementary Table S6).

Using the same method, interactions between APJ and β-arrestin 2 were determined by BRET. The BRET signals for all six peptide ligands increased dose-response dependently and peaked after about 15 min of treatment (Figures 7A–F). We also observed BRET signal in HEK293 cells, which expressed β-arrestin2 Rluc and APJ Venus labeled after stimulation with six peptide, resulting in a dose-dependent form. The BRET signal induced by Apelin-17 was significantly higher than the signals induced by the other peptide ligands (Figure 7G). The logEC_50_ values of the six agonists were −6.813 ± 0.091, −6.72 ± 0.218, −8.333 ± 0.157, −6.708 ± 0.248, −7.175 ± 0.14, and −7.878 ± 0.284, respectively.

### BRET Determination of Interactions Between β-arrestin 1/2 and β_2_-Adaptin Induced by APJ

**FIGURE 8:**
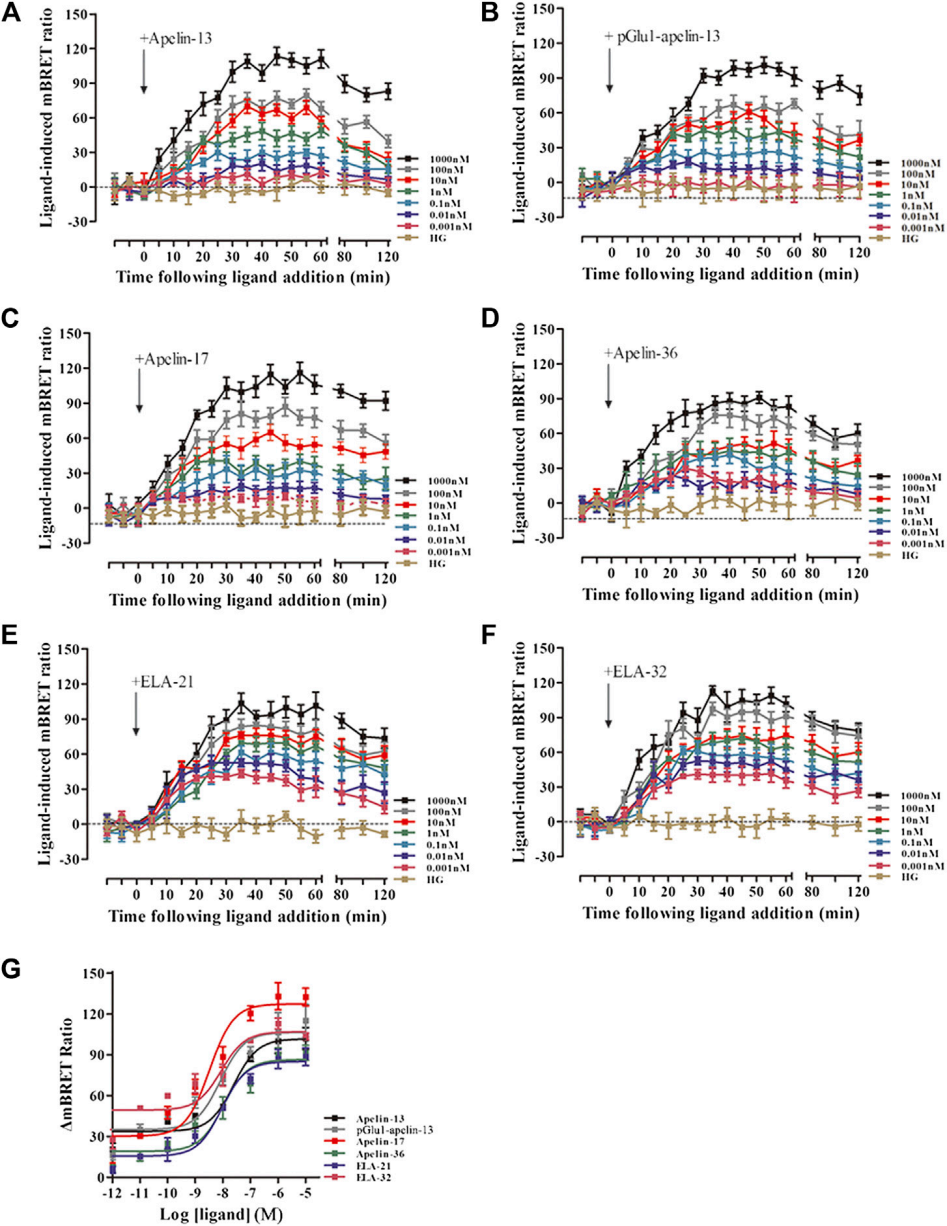
The time-response curve **(A–F)** and dose-response curve **(G)** of interactions between β-arrestin1 and AP2 induced by APJ upon activation by the six agonists in different concentrations, obtained from real-time dynamic BRET determination. *p* < 0.05 was considered as statistically significant. Control group (HG, Cells were stimulated with high glucose). The BRET assay to measure the six peptide (0.001–1,000 nM) induced concentration-response curves of β-arrestin 1 and β2-adaptin recruitment to APJ at 30 min post-stimulation **(G)**. The data represent means ± SEM of four independent experiments. Statistical analysis (Supplementary Table S7).

The real-time, dynamic determination of interactions of APJ with β-arrestin 1 and the β2-adaptin by BRET. HEK293 cells co-transfected with APJ, β-arrestin 1-Rluc, and β_2_-adaptin-EYFP were stimulated with the six peptide ligands at 0.001–1,000 nM concentration. All six peptide ligands increased the interaction between β-arrestin 1 and AP2 in a dose-dependent manner, and the signal reached its peak after 30 min of treatment with six peptide ligands (Figures 8A–F). Next, we used the BRET assay to measure the six peptide induced concentration-response curves of β-arrestin 1 and β2-adaptin recruitment to APJ at 30 min after stimulation. Six peptide caused a robust concentration-dependent. The BRET signal induced by Apelin-17 was significantly higher than that induced by the other peptide ligands (Figure 8G).

**FIGURE 9:**
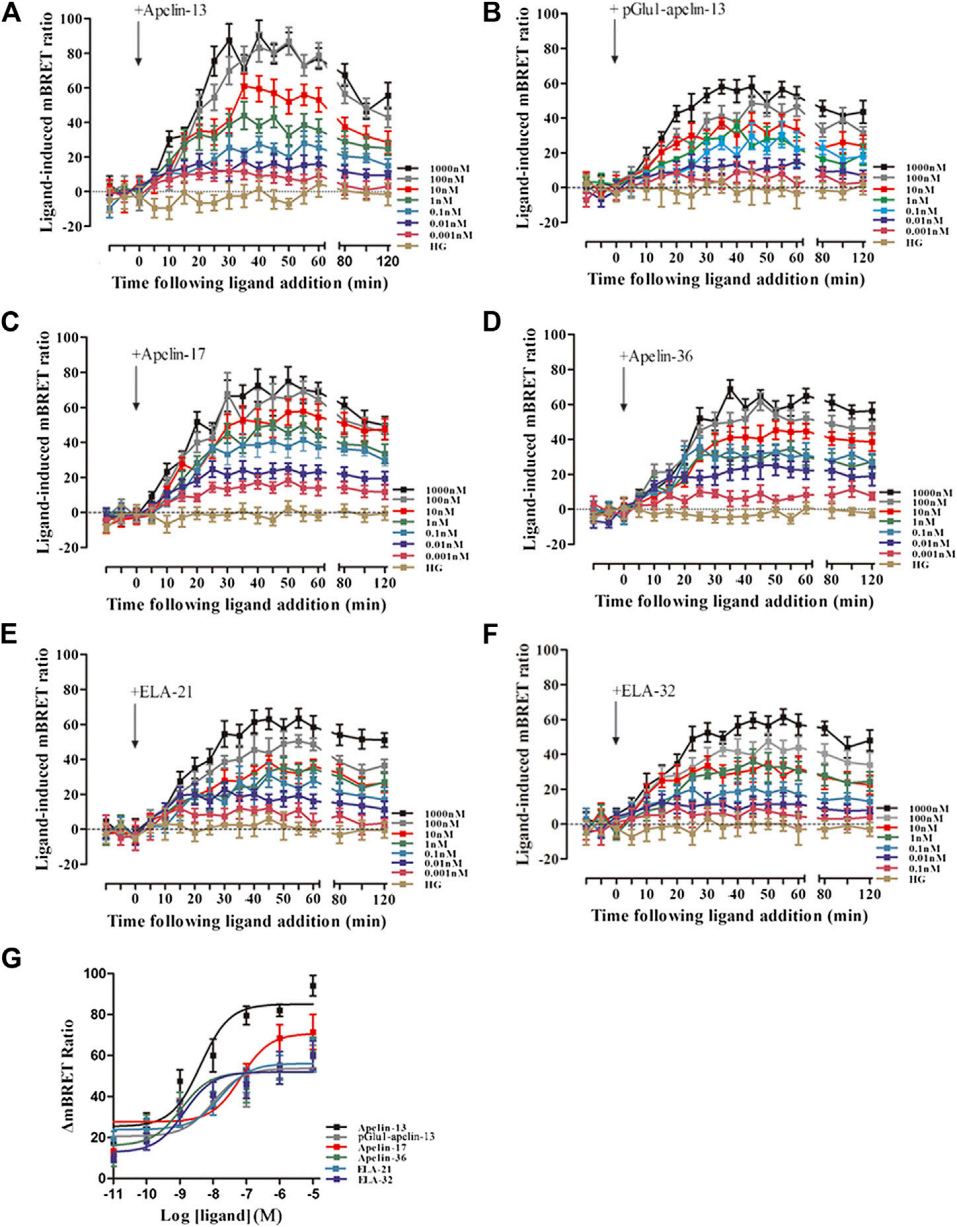
The time-response curve **(A–F)** and dose-response curve **(G)** of interactions between β-arrestin 2 and AP2 induced by APJ upon activation by the six agonists in different concentrations, obtained from real-time dynamic BRET determination. Control group (HG, Cells were stimulated with high glucose). *p* < 0.05 was considered as statistically significant. The BRET assay to measure the six peptide (0.001–1,000 nM) induced concentration-response curves of β-arrestin 2 and β2-adaptin recruitment to APJ at 30 min post-stimulation **(G)**. The data represent means ± SEM of four independent experiments. Statistical analysis (Supplementary Table S8).

Using the same method, the interactions between β-arrestin 2 and AP2 induced by APJ upon stimulation with the six peptide ligands were investigated. When HEK293 cells co-transfected with APJ, β-arrestin 2 -Rluc, and β_2_-adaptin-EYFP were stimulated with the six peptide ligands, all the BRET signals increased dose-dependently and peaked after 30 min of treatment (Figures 9A–F). HEK293 cells co-transfected with APJ, β-arrestin2 and AP2 were stimulated with different concentrations of six peptide peptides (0.01–10,000 nM) for 30 min to demonstrate their dose dependent characteristics. However, the BRET signal induced by Apelin-13 was significantly higher than that induced by the other peptide ligands (Figure 9G).

### Biases of the Ligands for Different Signal Transduction Pathways

**FIGURE 10:**
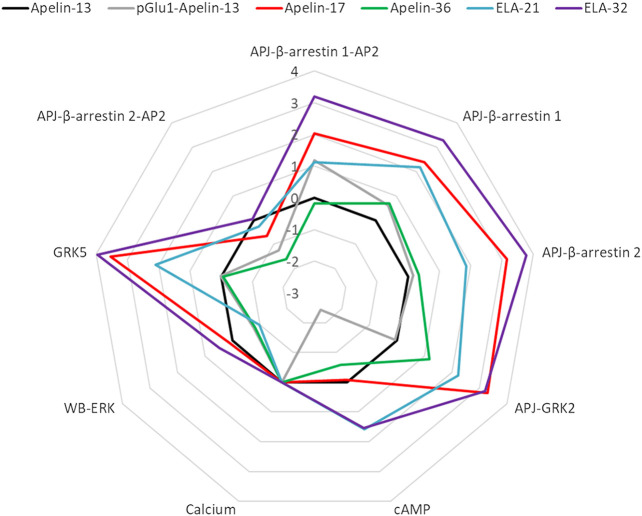
Web chart of Log (bias factor) of the six peptide ligands on eight pathways. Web of bias illustrating distinctions in the pattern of signaling of six peptide ligands at APJ. The web of bias plots ΔΔlog (*τ*/KA) values for each ligand and for every signaling pathway tested.

Finally, bias factors for the peptide ligands on the different signal transduction pathways were calculated (Table 3). The Apelin-13 was chosen as the reference ligand, and intracellular calcium release was selected as the reference pathway. The Log (Bias) values of each peptide ligand for each pathway are listed in Figure 10.

**TABLE 3:** Bias factor and 95% confidence interval of the peptide ligand.

	Apelin-13	pGlu_1_-Apelin-13	Apelin-17	Apelin-36	ELA-21	ELA-32
Ca^2+^						
log (*τ*/*K*_A_)	−0.2	−0.8	−0.9	−0.6	−1.8	−2.1
Δlog (*τ*/*K*_A_)	0.0	−0.6	−0.7	−0.4	−1.6	−1.9
Log (Bias)	0.0	0.0	0.0	0.0	0.0	0.0
Bias factor	1.0	1.0	1.0	1.0	1.0	1.0
WB-ERK						
log (*τ*/*K*_A_)	0.0 ± 0.2	−1.3 ± 0.2	−0.5 ± 0.2	−1.2 ± 0.2	−2.5 ± 0.2	−1.3 ± 0.2
Δlog (*τ*/*K*_A_)	0.0 ± 0.2	−1.3 ± 0.2	−0.6 ± 0.2	−1.2 ± 0.2	−2.6 ± 0.2	−1.4 ± 0.2
Log (Bias)	0.0	−0.8 ± 0.3	0.1 ± 0.3^a^	−0.8 ± 0.3	−1.0 ± 0.3	0.5 ± 0.3
Bias factor	1.0	0.2	1.3	0.1	0.1	3.0
GRK5						
log (*τ*/*K*_A_)	−2.4 ± 0.3	−2.9 ± 0.3	0.5 ± 0.3	−2.8 ± 0.3	−1.9 ± 0.3	−0.3 ± 0.3
Δlog (*τ*/*K*_A_)	0.0 ± 0.4	−0.5 ± 0.4	2.8 ± 0.4	−0.4 ± 0.4	0.5 ± 0.4	2.1 ± 0.4
Log (Bias)	0.0	0.0 ± 0.5^a^	3.5 ± 0.5	−0.1 ± 0.5	2.1 ± 0.5	4.0 ± 0.5
Bias factor	1.0	1.0	3,503.2	0.9	124.1	8,921.8
APJ-β-arrestin1-AP2						
log (*τ*/*K*_A_)	−1.4 ± 0.1	−0.8 ± 0.1	−0.1 ± 0.1	−1.9 ± 0.1	−1.9 ± 0.1	−0.1 ± 0.1
Δlog (*τ*/*K*_A_)	0.0 ± 0.2	0.6 ± 0.2	1.3 ± 0.2	−0.5 ± 0.2	−0.5 ± 0.2	1.3 ± 0.2
Log (Bias)	0.0	1.2 ± 0.3	2.0 ± 0.3	−0.2 ± 0.3^a^	1.1 ± 0.3	3.2 ± 0.3
Bias factor	1.0	15.0	109.4	0.7	13.2	1,580.1
APJ-β-arrestin1						
log (*τ*/*K*_A_)	−2.4 ± 0.1	−2.4 ± 0.1	−0.8 ± 0.1	−2.1 ± 0.1	−1.9 ± 0.1	−1.0 ± 0.1
Δlog (*τ*/*K*_A_)	0.0 ± 0.1	0.0 ± 0.1	1.7 ± 0.1	0.3 ± 0.1	0.6 ± 0.1	1.4 ± 0.1
Log (Bias)	0.0	0.6 ± 0.2	2.4 ± 0.2	0.7 ± 0.2	2.2 ± 0.2	3.3 ± 0.2
Bias factor	1.0	4.0	243.0	4.8	146.2	1978.4
APJ-β-arrestin 2						
log (*τ*/*K*_A_)	−2.4 ± 0.1	−2.8 ± 0.1	0.1 ± 0.1	−2.4 ± 0.1	−2.1 ± 0.1	−0.4 ± 0.1
Δlog (*τ*/*K*_A_)	0.0 ± 0.1	−0.4 ± 0.1	2.5 ± 0.1	0.0 ± 0.1	0.3 ± 0.1	1.9 ± 0.1
Log (Bias)	0.0	0.2 ± 0.2^a^	3.2 ± 0.2	0.3 ± 0.2	1.9 ± 0.2	3.8 ± 0.2
Bias factor	1.0	1.4	1,468.5	2.2	73.6	6,138.2
APJ-GRK2						
log (*τ*/*K*_A_)	−2.1 ± 0.2	−2.8 ± 0.2	0.4 ± 0.2	−1.3 ± 0.2	−1.5 ± 0.2	−0.8 ± 0.2
Δlog (*τ*/*K*_A_)	0.0 ± 0.3	−0.6 ± 0.3	2.6 ± 0.3	0.8 ± 0.3	0.6 ± 0.3	1.3 ± 0.3
Log (Bias)	0.0	−0.1 ± 0.4^a^	3.3 ± 0.4	1.2 ± 0.4	2.2 ± 0.4	3.2 ± 0.4
Bias factor	1.0	0.9	1972.2	15.6	169.0	1,604.0
cAMP						
log (*τ*/*K*_A_)	0.0 ± 0.1	−3.0 ± 0.1	−0.8 ± 0.1	−1.0 ± 0.1	−0.1 ± 0.1	−0.4 ± 0.1
Δlog (*τ*/*K*_A_)	0.0 ± 0.2	−3.0 ± 0.2	−0.8 ± 0.2	−1.0 ± 0.2	0.0 ± 0.2	−0.3 ± 0.2
Log (Bias)	0.0	−2.4 ± 0.3	−0.1 ± 0.3^a^	−0.6 ± 0.3	1.6 ± 0.3	1.5 ± 0.3
Bias factor	1.0	0.0037	0.8	0.3	38.0	34.2
APJ-β-arrestin2-AP2						
log (*τ*/*K*_A_)	−0.2 ± 0.4	−2.0 ± 0.4	−1.6 ± 0.4	−2.2 ± 0.4	−2.1 ± 0.4	−2.0 ± 0.4
Δlog (*τ*/*K*_A_)	0.0 ± 0.5	−1.8 ± 0.5	−1.4 ± 0.5	−2.0 ± 0.5	−1.9 ± 0.5	−1.8 ± 0.5
Log (Bias)	0.0	−1.3 ± 0.6	−0.7 ± 0.6	−1.6 ± 0.6	−0.3 ± 0.6^a^	0.0 ± 0.6^a^
Bias factor	1.0	0.06	0.2	0.02	0.5	1.1

^a^ Statistically insignificant.

With the exception of Apelin-36, all the ligands exhibited significant biases for the other pathways over the calcium release pathway. The Elabela-derived ligand, Elabela-32, showed the greatest bias for the other pathways over the calcium pathway; notably, it induced APJ–GRK5 and APJ–β-arrestin 2 interactions >6,000-fold relative to its induction of calcium release, while it induced β-arrestin 1–AP2, APJ–β-arrestin 1, and APJ–GRK2 interactions >1,500-fold more strongly than calcium release.

The Apelin-derived ligand Apelin-17 showed a similar bias profile to Elabela-32, but the activation of signaling pathways was weaker overall (Log (Bias): GRK5: 3.5 vs. 4.0; APJ-beta-arrestin1-AP2: 2.0 vs. 3.2; APJ-beta-arrestin1: 2.4 vs. 3.3; APJ-beta-arrestin2: 3.2 vs. 3.8; GRK2: 3.3 vs. 3.2). Apelin-17 showed no bias for the cAMP and ERK pathways, but had comparable effects on APJ–GRK5, APJ–GRK2, and APJ–β-arrestin 2 interactions to Elabela-32. It induced APJ–β-arrestin 1and APJ–β-arrestin 1–AP2 interactions >100-fold more strongly than it induced calcium release.

The Elabela-derived peptide ligand Elabela-21 showed a similar bias profile as Apelin-17 but displayed the strongest activation of the cAMP pathway among all the ligands [38-fold higher than its activation of calcium release, which was slightly higher than that of Elabela-32 (34.2-fold)]. Elabela-21 also showed the weakest activation of the ERK pathway (0.1-fold) among the six APJ ligands.

The Apelin-36 and pGlu_1_-apelin-13 ligand showed negative biases toward the pathway APJ-β-arrestin2-AP2 (0.02-fold and 0.06-fold, respectively). The pGlu_1_-apelin-13 also showed a strong negative bias toward the cAMP pathway (0.0037-fold). These two ligands displayed relatively weak biases on other pathways.

## Discussion

GPCR ligands were traditionally thought to act equally on all the downstream signaling pathways of the GPCRs, including the multiple signaling pathways mediated by G proteins and β-arrestin. However, the studies showed that the activation of different signal pathways is not necessarily equivalent or proportional, and ligands usually exhibit specific biases for certain pathways, such that when one pathway is activated, another pathway may be activated only weakly, or even inhibited (Gurwitz et al., 1994; Kim et al., 2008) Such biased ligands are able to differentially activate certain pathway(s) mediated by G proteins or β-arrestin, and exhibit higher selectivity than the canonical balanced ligands. These ligands are therefore very meaningful for the design and discovery of novel drugs. Currently, drug candidates with high biases for G proteins or β-arrestin are under development, and are expected to possess high selectivity and minimal adverse effects. For instance, *μ* opioid receptor (MOR) agonists are commonly used to treat severe pain, but they can cause serious adverse effects including drug tolerance, respiratory depression, and constipation. Such adverse effects have been linked to the activation of β-arrestin signaling. Oliceridine (TRV-130) is a novel *μ* opioid agonist, recently approved in the FDA for the treatment of acute pain in adults. The interaction between oliceridine and opioid receptor is selective for G protein pathway, and the effect of β - arrestin recruitment is low, which may lead to fewer opioid related adverse events (Markham, 2020).

On the other hand, activation of the β-arrestin pathway by angiotensin II receptor 1 (AT1R) may promote cardiomyocyte contraction and improve cardiovascular pump function, and may therefore benefit patients with acute heart failure (Whalen et al., 2011) In human studies, the first discovery of novel biased APJ agonist MM07, as a biased agonist of APJ, it can preferentially stimulate G protein pathway and avoid activating harmful β-arrestin dependent pathway by selectively stimulating vasodilation and positive inotropic effect, so as to improve clinical efficacy (Brame et al., 2015). Elabela also that binds to APJ, activates the Gαi1 and β-arrestin-2 signaling pathways (Murza et al., 2016). The Apelin-36-[L28A] and Apelin-36-[L28C(30kDa-PEG)] bind to the APJ with nanomolar affinities. The data provide evidence that these peptides are G protein biased APJ agonists (Nyimanu et al., 2019).

Our group (Chen et al., 2014) determined that APJ with a S348A mutation is unable to activate the β-arrestin pathway and therefore exhibits a bias for G protein signaling. Conversely, with respect to β-arrestin dependent signal transduction, Phe255 and Trp259 in rat APJ (corresponding to Phe257 and Trp263 in human APJ) are critical for rapid receptor internalization induced by binding of Apelin-17 or pGlu_1_-apelin-13 to APJ (Iturrioz et al., 2010). Recently, our results indicate that Apelin-36, Apelin-17, Apelin-13, Elabela-32, and Elabela-21 peptides act on different phosphorylation sites at the C-terminal of APJ to regulate APJ signal transduction and cause different biological effects. However, different peptides act on different structural parts of APJ to produce different signal transduction pathways, laying the foundation for studying the relationship between structure and function.

In human and rat plasma, Apelin-17 and pGlu_1_-apelin-13 are considered as a predominant isoform and those are secreted from atria and adipose tissues, the main source of plasma Apelin (Kuba et al., 2019). Biased signal transduction can occur in response to ligands. El Messari et al. (2004) have reported that compared with Apelin-13, Apelin-17 is better able to recruit β-arrestin and internalize the receptor. Meanwhile, studies have shown that loss of the C-terminal phenylalanine can lead to a bias in G protein signaling (Ceraudo et al., 2014). These studies, therefore, suggest that longer length peptides are able to reach a binding pocket that is not accessible to the shorter Apelin-13 isoform to induce β-arrestin recruitment and internalization. Of interest, our results also showed that Apelin-36 did not show β-arrestins biased signal transduction. This suggests that the relationship between peptide length and signal bias is more complex. On the contrary, only longer ligands can enter the binding pocket that induces β-arrestins recruitment. This is further supported by longer length Elabela peptide also possessing β-arrestin bias (Yang et al., 2017). Elabela is more strongly positively charged than Apelin and also displays higher binding affinities to the receptor at corresponding fragment sizes, lending support to an ionic interaction as critical to binding (Langelaan and Rainey, 2010; Kuba et al., 2019).

The findings reported in the present study are consistent with this, in that Elabela-32 had the highest binding affinities to the APJ. Elabela is downregulated in human disease and rodent pulmonary arterial hypertension (PAH) models, and exogenous peptide can reduce the severity of cardiopulmonary remodeling and function in PAH in rats (Yang et al., 2017). In our competitive binding assay, we found that [^125^ I] apelin-13 binds to the APJ with high affinity, while cold Apelin-36 (IC50) was 1.9 nM. This result is the same as the binding affinity for APJ of Apelin-36 (IC50 = 2.3 nM) reported by Fan et al. (2003). We used [^125^ I]-apelin-13 as the reference ligand to test the binding ability of these peptides. Compared with Elabela-32 and Elabela-21, the binding ability of the latter was weaker. The results showed that Elabela peptide did not have the same binding site as Apelin on APJ. These studies broaden our knowledge of the relationship between receptor structure and signaling bias. Couvineau et al. evaluated the ability of two human Elabela (Elabela-32 and Elabela 21) and an apelin (K17F) to displace the binding of 125I-pE13F to the APJ, The Elabela-32 and Elabela 21and apelin (K17F) peptides had pKi values in the subnanomolar range, at 9.86, 9.49 and 10.18, respectively. However, our results that the pKi values obtained for the Apelin-13, Apelin-17, Apelin-36, Elabela-32 and Elabela 21 were in the subnanomolar range at 8.08, 8.33, 8.76, 8.87 and 8.36 (Table 1), respectively. We obtained similar results with Couvineau et al., and agreed that the structural features of Elabela and Apelin were different, resulting in different modes of binding of these endogenous ligands to the APJ (Couvineau et al., 2020).

However, for drug design and development, the more attractive approach would be the engineering of ligands exhibiting biases for various pathways, which may selectively activate only one or a few desired pathways downstream of a particular receptor without stimulating other undesired pathways, such as those that lead to adverse effects. Murza et al. APJ can initiate ERK1/2 signal transduction through G protein dependent and - β-arrestin dependent pathways. G protein dependent signal shows early and transient response, while β-arrestin dependent signal is late and persistent (DeWire et al., 2007; Kuba et al., 2019). We use the early phase ERK activation at 5 min stimulation as one of the important indicators of G protein dependent signal transduction pathway. It is consistent with the results of Ceraudo et al., which confirmed that G protein dependent on ERK pathway by using pertussis toxin (Ceraudo et al., 2014).

For GPCR, agonist stimulation leads to GRKs mediated receptor phosphorylation, followed by binding of β-arrestin to phosphorylated receptors. GRKs and β-arrestins are important signal transducers. Many biological functions of GPCR are mediated by β-arrestin dependent signal transduction. We also use clathrin as one of β-arrestin-dependent signaling indicators. The activities of six peptide ligands on APJ mediated G protein dependent and β-arrestin dependent signaling pathways were systematically studied.

The ligands had diverse preferences among the different pathways (Daniele et al., 2014); some ligands even exhibited a >1000-fold bias. For example, Elabela-32 strongly induced APJ–GRK5, APJ–GRK2, APJ–β-arrestin 2, and APJ–β-arrestin 1–AP2 interactions, but showed very weak effects on calcium mobilization and ERK activation, indicating that Elabela-32 had a strong bias for β-arrestin dependent signaling. Interestingly, Murza et al. (2016) showed that Elabela (19–32) that binds to APJ, activates the Gαi1 and β-arrestin 2 signaling pathways, and induces receptor internalization similarly to its parent endogenous peptide. Recently, Ho et al. demonstrated that Elabela-APJ signaling axis may offer a new paradigm for the treatment of common pregnancy-related complications, including Preeclampsia (Ho et al., 2017). We will further examine whether Elabela-32 biased signaling is involved in the mechanism of Preeclampsia. We measured the six peptide induced concentration-response curves of β-arrestin 1, β-arrestin 2 and β2-adaptin recruitment to APJ. Six peptide caused a concentration-dependent characteristics. The BRET signal induced by other peptide ligands was significantly lower than that induced by Apelin-17. However, Apelin-17 was biased toward β-arrestin dependent biased signaling and showed weak activation of G protein dependent pathways (calcium, cAMP, and ERK). Based on these findings, The results Apelin-17 may be as a β-arrestin dependent signaling which may play an important role in blood pressure (BP) decrease, in agreement with their effects on the β-arrestin-dependent ERK1/2 activation and not the Gαi-dependent signaling may participate in Apelin-K17F [a 17-amino acid chain stretching from lysine (1) to phenylalanine (17) of the Apelin sequence] induced BP decrease (Ceraudo et al., 2014). El Messari et al. (2004) have also reported that Apelin-17 is better able to recruit β-arrestin and internalize the receptor than Apelin-13. Therefore, Elabela-32, Apelin-17, or their derivatives could be used to selectively activate β-arrestin dependent signaling pathways while minimizing the activation of G protein dependent pathways to treat disease while avoiding unwanted effects on other pathways. Elabela-32 and Apelin-17 favor β-arrestin-dependent biased signal transduction, and exhibit weak activation of G protein-dependent pathways. According to our research, elabela-32 and apelin-17, as β-arrestin-dependent signal transduction pathways, may play an important role in lowering blood pressure and enhancing cardiac function through vasodilation.

On the other hand, Apelin-36 showed similar activity profiles to the reference ligand, Apelin-13, and did not display significant biases for G protein dependent or β-arrestin dependent signaling pathways. Lee et al. (2010) demonstrated that Apelin-13-mediated internalization could be rapidly reversed when washed out, whereas Apelin-36 resulted in more prolonged receptor internalization.

Moreover, among the G protein dependent pathways (calcium, cAMP, and ERK), the peptide ligands still exhibited distinct activation profiles. Compared with the reference pathway (calcium release), Elabela-32 and Elabela-21 displayed much stronger activity on the cAMP pathway, whereas pGlu_1_-apelin-13 showed very weak activity. With respect to the ERK pathway, Elabela-32 showed stronger activity, while its truncated peptide Elabela-21, pGlu_1_-apelin-13, and Apelin-36 showed weaker activity. Thus, it is possible to selectively activate one of these pathways without affecting the others to avoid potential adverse effects. For example, Elabela-21 may be used to selectively stimulate cAMP signaling over calcium release and ERK activation; also, one could use pGlu1-apelin-13 to selectively induce calcium release while minimizing effects on the cAMP and ERK pathways.

Furthermore, Elabela-21 and pGlu_1_-apelin-13 exhibited very distinct activities on the G protein dependent pathways, but showed very weak effects on β-arrestin dependent pathways. Elabela-21 and pGlu_1_-apelin-13 shows a signal transduction pathway similar to that of APJ agonist MM07. They preferentially stimulate G protein pathway and avoid activating harmful β-arrestin dependent pathway, thus selectively stimulate vasodilation and positive inotropic effect, and produce protective response to myocardial hypertrophy.

However, the Apelin and Elabela proteins constitute a spatiotemporal double-ligand system that controls APJ signal transduction (Chen et al., 2020) and function. The ligands exhibited distinct signaling profiles. These data provide that the six peptide ligands of APJ exhibit different biases for activation of various APJ downstream signaling pathways. Elabela-32 induced β-arrestin dependent signaling, but showed very weak effects on G protein signaling, indicating that Elabela-32 had a bias for β-arrestin dependent signaling. At the same time, Apelin-17 was biased toward β-arrestin dependent signaling. Eabela-21 and pGlu_1_-apelin-13 exhibited very distinct activities on the three G protein dependent pathways (calcium, cAMP, and ERK). On the other hand, Apelin-36 showed similar activity profiles to the reference ligand, Apelin-13, and did not display significant biases for G protein dependent or β-arrestin dependent signaling pathways. However, further research on the bias signal in the Apelin and Elabela dual-ligand system of APJ receptors will help new treatment methods for heart and other diseases.

The activity profiles of the peptide ligands suggest that these ligands may serve as therapeutic compounds that could be used to stimulate one signaling pathway without significant effects on the other pathways. The distinct activities and biases of the Apelin/Elabela-derived peptides demonstrates that truncations or simple mutations can significantly change the activity profiles of the ligands. This observation suggests that the activity and selectivity of peptide ligands on certain pathways could be improved through structural modifications. Furthermore, the ligand activity and bias profiles reported here also provide a starting point for artificially modulating APJ signal transduction, and will therefore be meaningful for both mechanistic studies and drug development. However, *in vivo* studies are needed to further assess the importance of the reported ligand activity and bias profiles.

## Data Availability

The original contributions presented in the study are included in the article/Supplementary Material, further inquiries can be directed to the corresponding author.

## Abbreviations

AP2, β2 adaptin-EYF; BRET, bioluminescence resonance energy transfer; cAMP, cyclic AMP, Cyclic adenosine monophosphate; CAMYEL, cAMP sensor using YFP-Epac-RLuc; ELA, Elabela; ERK, extracellular signal regulated kinases; EYFP, Enhanced Yellow Fluorescent Protein; GH, High glucose; GPCR, G protein-coupled receptor ; GRKs, G protein-coupled receptor kinases; HEK, Human embryonic kidney; RLuc, Renilla luciferase.
