# Supplementary material for: The Effects of Apelin and Elabela Ligands on Apelin Receptor Distinct Signaling Profiles
Source: Front Pharmacol. 2021 Mar 4;12:630548. doi: 10.3389/fphar.2021.630548 (PMC7970304; doi:10.3389/fphar.2021.630548)
Supplement: Supplementary file 1 [file datasheet1.doc]

**The effects of Apelin and Elabela ligands on Apelin receptor distinct signaling profiles**

Yunlu Jiang^1^#, Maocai Yan^2^#, Chunmei Wang^1^ ,Qinqin Wang^1^, Xiaoyu Chen^3^, Rumin Zhang^1^, Lei Wan^1^ , Bingyuan Ji ^1^, Bo Dong ^4^, Huiyun Wang^2^ and Jing Chen ^1,5^ *

1 Neurobiology Key Laboratory of Jining Medical University in Colleges of Shandong, Jining Medical University, Jining, China.

2 School of Pharmacy, Jining Medical University, Shandong China.

3 Department of physiology, Shandong First Medical University, Shandong China.

4 Shandong Provincial Hospital Affiliated to Shandong University, Jinan, China

5 Division of Biomedical Sciences, Warwick Medical School, University of Warwick, Coventry, CV4 7AL, United Kingdom.

**Supplementary Material**

**1, Statistical analysis: Table S1-Table S7**

1. Intracellular cAMP production induced by the six agonists (Supplementary Figure 2).

Statistical analysis (Table S1)

| *vs* HG | 1000 nM | 100 nM | 10n M | 1 nM | 0.1 nM | 0.01 nM | 0.001 nM |
| --- | --- | --- | --- | --- | --- | --- | --- |
| Apelin13 | ^***^*P*<0.001 | ^***^*P*<0.001 | ^***^*P*<0.001 | ^**^*P*<0.01 | *P>*0.05 | *P>*0.05 | *P>*0.05 |
| pGlu_1_-apelin-13 | ^***^*P*<0.001 | ^***^*P*<0.001 | ^***^*P*<0.001 | ^***^*P*<0.001 | ^*^*P*<0.05 | *P>*0.05 | *P>*0.05 |
| Apelin-17 | ^***^*P*<0.001 | ^***^*P*<0.001 | ^***^*P*<0.001 | ^**^*P*<0.01 | ^*^*P*<0.05 | *P>*0.05 | *P>*0.05 |
| Apelin-36 | ^***^*P*<0.001 | ^***^*P*<0.001 | ^***^*P*<0.001 | ^**^*P*<0.01 | *P>*0.05 | *P>*0.05 | *P>*0.05 |
| Ela-21 | ^***^*P*<0.001 | ^***^*P*<0.001 | ^***^*P*<0.001 | ^**^*P*<0.01 | *P>*0.05 | *P>*0.05 | *P>*0.05 |
| Ela-32 | ^***^*P*<0.001 | ^***^*P*<0.001 | ^***^*P*<0.001 | ^***^*P*<0.001 | *^*^P<*0.05 | *P>*0.05 | *P>*0.05 |

1. The intracellular Calcium production induced by the six agonists (Supplementary Figure 3)

Statistical analysis (Table S2)

| *vs* HG | 1000 nM | 100 nM | 10 nM | 1 nM | 0.1 nM | 0.01 nM | 0.001 nM |
| --- | --- | --- | --- | --- | --- | --- | --- |
| Apelin13 | ^***^*P*<0.001 | ^**^*P*<0.001 | ^**^*P*<0.001 | *P>*0.05 | *P>*0.05 | *P>*0.05 | *P>*0.05 |
| pGlu_1_-apelin 13 | ^***^*P*<0.001 | ^**^*P*<0.001 | ^**^*P*<0.001 | *P>*0.05 | *P>*0.05 | *P>*0.05 | *P>*0.05 |
| Apelin-17 | ^***^*P*<0.001 | ^**^*P*<0.001 | ^**^*P*<0.001 | ^*^*P*<0.05 | *P*>0.05 | *P>*0.05 | *P>*0.05 |
| Apelin-36 | ^***^*P*<0.001 | ^**^*P*<0.01 | ^*^*P*<0.05 | ^*^*P*<0.05 | *P>*0.05 | *P>*0.05 | *P>*0.05 |
| Ela-21 | ^***^*P*<0.001 | ^***^*P*<0.001 | ^*^*P*<0.05 | *P>*0.05 | *P>*0.05 | *P>*0.05 | *P>*0.05 |
| Ela-32 | ^***^*P*<0.001 | ^**^*P*<0.01 | ^*^*P*<0.05 | *P>*0.05 | *P>*0.05 | *P>*0.05 | *P>*0.05 |

1. The time-response curve of interactions between APJ and GRK2 induced by the six agonists of different concentrations, obtained from real-time dynamic BRET determination (Supplementary Figure 5)

Statistical analysis (Table S3)

APJ-GRK_2_

| *vs* HG | 1000 nM | 100 nM | 10 nM | 1 nM | 0.1 nM | 0.01 nM | 0.001 nM |
| --- | --- | --- | --- | --- | --- | --- | --- |
| Apelin13 | ^***^*P*<0.001 | ^***^*P*<0.001 | ^**^*P*<0.01 | ^*^*P*<0.05 | *P>*0.05 | *P>*0.05 | *P>*0.05 |
| pGlu_1_-apelin-13 | ^***^*P*<0.001 | ^***^*P*<0.001 | ^***^*P*<0.001 | ^***^*P*<0.001 | *P>*0.05 | *P>*0.05 | *P>*0.05 |
| Apelin-17 | ^***^*P*<0.001 | ^***^*P*<0.001 | ^***^*P*<0.001 | ^*^*P*<0.05 | *P>*0.05 | *P>*0.05 | *P>*0.05 |
| Apelin-36 | ^***^*P*<0.001 | ^***^*P*<0.001 | ^**^*P*<0.001 | ^*^*P*<0.05 | *P>*0.05 | *P>*0.05 | *P>*0.05 |
| Ela-21 | ^***^*P*<0.001 | ^***^*P*<0.001 | ^**^*P*<0.001 | *P>*0.05 | *P>*0.05 | *P>*0.05 | *P>*0.05 |
| Ela-32 | ^***^*P*<0.001 | ^***^*P*<0.001 | ^***^*P*<0.001 | ^**^*P*<0.01 | *P>*0.05 | *P>*0.05 | *P>*0.05 |

1. The time-response curve and dose-response curve of interactions between APJ and GRK5 induced by the six agonists of different concentrations, obtained from real-time dynamic BRET determination (Figure 5)

Statistical analysis (Table S4)

_APJ-GRK_5

| *vs* HG | 1000 nM | 100 nM | 10 nM | 1 nM | 0.1 nM | 0.01 nM | 0.001 nM |
| --- | --- | --- | --- | --- | --- | --- | --- |
| Apelin13 | ^***^*P*<0.001 | ^***^*P*<0.001 | ^***^*P*<0.001 | ^***^*P*<0.001 | *^**^P<*0.01 | *P>*0.05 | *P>*0.05 |
| pGlu_1_-apelin-13 | ^***^*P*<0.001 | ^***^*P*<0.001 | ^*^*P*<0.05 | ^*^*P*<0.05 | *P>*0.05 | *P>*0.05 | *P>*0.05 |
| Apelin-17 | ^***^*P*<0.001 | ^***^*P*<0.001 | ^***^*P*<0.001 | ^***^*P*<0.001 | *P>*0.05 | *P>*0.05 | *P>*0.05 |
| Apelin-36 | ^***^*P*<0.001 | ^***^*P*<0.001 | ^**^*P*<0.001 | ^*^*P*<0.05 | *P>*0.05 | *P>*0.05 | *P>*0.05 |
| Ela-21 | ^***^*P*<0.001 | ^***^*P*<0.001 | ^**^*P*<0.001 | *P>*0.05 | *P>*0.05 | *P>*0.05 | *P>*0.05 |
| Ela-32 | ^***^*P*<0.001 | ^***^*P*<0.001 | ^***^*P*<0.001 | ^*^*P*<0.05 | *P>*0.05 | *P>*0.05 | *P>*0.05 |

1. The time-response curve and dose-response curve of interactions between APJ and β-arrestin1 induced by the six agonists in different concentrations, obtained from real-time dynamic BRET determination (Figure 6).

Statistical analysis (Table S5)

| *vs* HG | 1000 nM | 100 nM | 10 nM | 1 nM | 0.1 nM | 0.01 nM | 0.001 nM |
| --- | --- | --- | --- | --- | --- | --- | --- |
| Apelin13 | ^***^*P*<0.001 | ^***^*P*<0.001 | ^**^*P*<0.01 | ^*^*P*<0.05 | *P>*0.05 | *P>*0.05 | *P>*0.05 |
| pGlu_1_-apelin-13 | ^***^*P*<0.001 | ^***^*P*<0.001 | ^*^*P*<0.05 | *P>*0.05 | *P>*0.05 | *P>*0.05 | *P>*0.05 |
| Apelin-17 | ^***^*P*<0.001 | ^***^*P*<0.001 | ^**^*P*<0.01 | *P>*0.05 | *P>*0.05 | *P>*0.05 | *P>*0.05 |
| Apelin-36 | ^***^*P*<0.001 | ^***^*P*<0.001 | ^*^*P*<0.05 | *P>*0.05 | *P>*0.05 | *P>*0.05 | *P>*0.05 |
| Ela-21 | ^***^*P*<0.001 | ^***^*P*<0.001 | ^*^*P*<0.05 | *P>*0.05 | *P>*0.05 | *P>*0.05 | *P>*0.05 |
| Ela-32 | ^***^*P*<0.001 | ^***^*P*<0.001 | ^***^*P*<0.001 | ^*^*P*<0.05 | *P>*0.05 | *P>*0.05 | *P>*0.05 |

1. The time-response curve and dose-response curve of interactions between APJ and β-arrestin2 induced by the six agonists in different concentrations, obtained from real-time dynamic BRET determinations (Figure 7).

Statistical analysis (Table S6)

| *vs* HG | 1000 nM | 100 nM | 10 nM | 1 nM | 0.1 nM | 0.01 nM | 0.001 nM |
| --- | --- | --- | --- | --- | --- | --- | --- |
| Apelin13 | ^***^*P*<0.001 | ^***^*P*<0.001 | ^***^*P*<0.001 | ^**^*P*<0.01 | *^*^P<*0.05 | *P>*0.05 | *P>*0.05 |
| pGlu_1_-apelin-13 | ^***^*P*<0.001 | ^***^*P*<0.001 | ^***^*P*<0.001 | *^*^P<*0.05 | *P>*0.05 | *P>*0.05 | *P>*0.05 |
| Apelin-17 | ^***^*P*<0.001 | ^***^*P*<0.001 | ^***^*P*<0.001 | *^*^P<*0.05 | *^*^P<*0.05 | *P>*0.05 | *P>*0.05 |
| Apelin-36 | ^***^*P*<0.001 | ^***^*P*<0.001 | ^***^*P*<0.001 | ^*^*P*<0.05 | *P>*0.05 | *P>*0.05 | *P>*0.05 |
| Ela-21 | ^***^*P*<0.001 | ^***^*P*<0.001 | ^*^*P*<0.05 | *P>*0.05 | *P>*0.05 | *P>*0.05 | *P>*0.05 |
| Ela-32 | ^***^*P*<0.001 | ^***^*P*<0.001 | ^***^*P*<0.001 | ^***^*P*<0.001 | *P>*0.05 | *P>*0.05 | *P>*0.05 |

1. The dose-response curve (A-G) of interactions between β-arrestin1 and AP2 induced by APJ upon activation by the six agonists in different concentrations, obtained from real-time dynamic BRET determination (Figure 8)

Statistical analysis (Table S7)

| *vs* HG | 1000 nM | 100 nM | 10 nM | 1 nM | 0.1 nM | 0.01 nM | 0.001 nM |
| --- | --- | --- | --- | --- | --- | --- | --- |
| Apelin13 | ^***^*P*<0.001 | ^***^*P*<0.001 | ^***^*P*<0.001 | ^**^*P*<0.01 | *P>*0.05 | *P>*0.05 | *P>*0.05 |
| pGlu_1_-apelin-13 | ^***^*P*<0.001 | ^***^*P*<0.001 | ^***^*P*<0.001 | *^***^P<*0.001 | *^*^P<*0.05 | *P>*0.05 | *P>*0.05 |
| Apelin-17 | ^***^*P*<0.001 | ^***^*P*<0.001 | ^***^*P*<0.001 | *^**^P<*0.01 | *^*^P<*0.05 | *P>*0.05 | *P>*0.05 |
| Apelin-36 | ^***^*P*<0.001 | ^***^*P*<0.001 | ^***^*P*<0.001 | *^***^P<*0.001 | *^*^P<*0.05 | *P>*0.05 | *P>*0.05 |
| Ela-21 | ^***^*P*<0.001 | ^***^*P*<0.001 | ^***^*P*<0.001 | ^***^*P*<0.001 | ^***^*P*<0.001 | *^**^P<*0.01 | *^*^P<*0.05 |
| Ela-32 | ^***^*P*<0.001 | ^***^*P*<0.001 | ^***^*P*<0.001 | ^***^*P*<0.001 | *^**^P<*0.01 | *^**^P<*0.01 | *P>*0.05 |

1. The dose-response curve (A-G) of interactions between β-arrestin2 and AP2 induced by APJ upon activation by the six agonists in different concentrations, obtained from real-time dynamic BRET determination (Figure 9)

Statistical analysis (Table S8)

| *vs* HG | 1000 nM | 100 nM | 10 nM | 1 nM | 0.1 nM | 0.01 nM | 0.001 nM |
| --- | --- | --- | --- | --- | --- | --- | --- |
| Apelin13 | ^***^*P*<0.001 | ^***^*P*<0.001 | ^***^*P*<0.001 | ^***^*P*<0.001 | *P>*0.05 | *P>*0.05 | *P>*0.05 |
| pGlu_1_-apelin-13 | ^***^*P*<0.001 | ^***^*P*<0.001 | ^***^*P*<0.001 | *^***^P<*0.001 | *^*^P<*0.05 | *P>*0.05 | *P>*0.05 |
| Apelin-17 | ^***^*P*<0.001 | ^***^*P*<0.001 | ^***^*P*<0.001 | *^***^P<*0.001 | *^**^P<*0.01 | *P>*0.05 | *P>*0.05 |
| Apelin-36 | ^***^*P*<0.001 | ^***^*P*<0.001 | ^***^*P*<0.001 | *^***^P<*0.001 | *^***^P<*0.001 | *^*^P<*0.05 | *P>*0.05 |
| Ela-21 | ^***^*P*<0.001 | ^***^*P*<0.001 | ^***^*P*<0.001 | ^**^*P*<0.001 | ^*^*P*<0.05 | *P>*0.01 | *P>*0.05 |
| Ela-32 | ^***^*P*<0.001 | ^***^*P*<0.001 | ^***^*P*<0.001 | ^***^*P*<0.001 | *^**^P<*0.01 | *P>*0.05 | *P>*0.05 |

**2, Supplementary Figure 1**


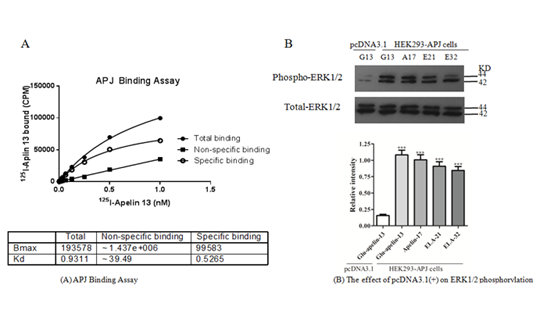


Supplementary Figure 1: (A) Saturation Binding Assay: Incubate 120 µl HEK-293/APJ membrane (20 µg/well) and 15 µl of [^125^I]- apelin 13 (2 fold dilution from 1 nM, 10 points) with/without 15 µl of cold Apelin 13 (100 nM final) in binding assay buffer at 300C for 2 hr. Stop the binding reaction, wash and dry the plates, add scintillation cocktail, and measure determine the radioactivity by Perkin Elmer 1450 MicroBeta TriLux Microplate Scintillation and Luminescence Counter.

(B) ERK1/2 phosphorylation in HEK293 cells with transient expression of pcDNA 3.1(+) (Control) or APJ, after 5-min stimulation by the pGlu_1_-apelin-13, Apelin-17 Elabela-21, and Elabela-32 peptide of 100 nM. Levels of p-ERK, total ERK were determined by western blot. The data represent means ± SEM of three independent experiments.

2, Supplementary Figure 2


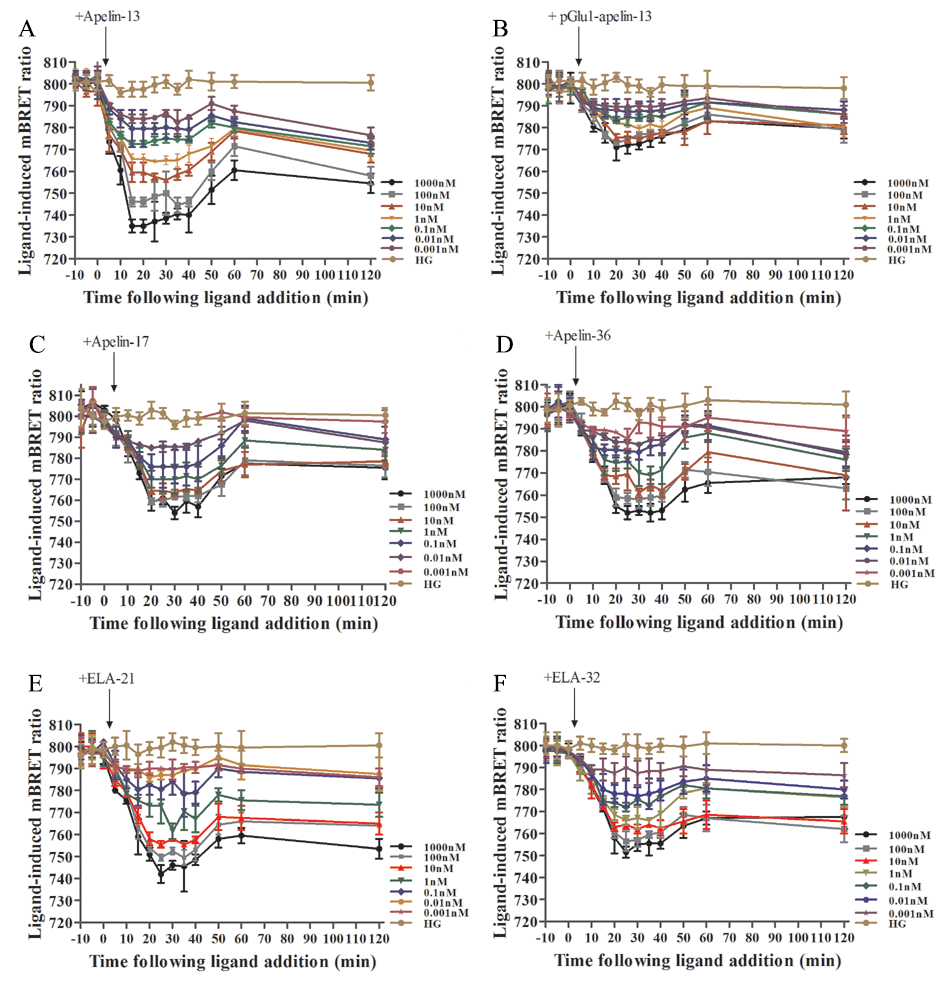


Supplementary Figure 2: (A-F) The cAMP time-response curve of the six agonists of various concentrations in HEK293 cells with stable expression of APJ. P<0.05 was considered as statistically significant. Control group (HG, Cells were stimulated with high glucose (Glucose Concentration: 4500 mg/L, HEPES, No Phenol Red). Data represent mean ± SEM from four independent experiments. Statistical analysis (Table S1)

3, Supplementary Figure 3


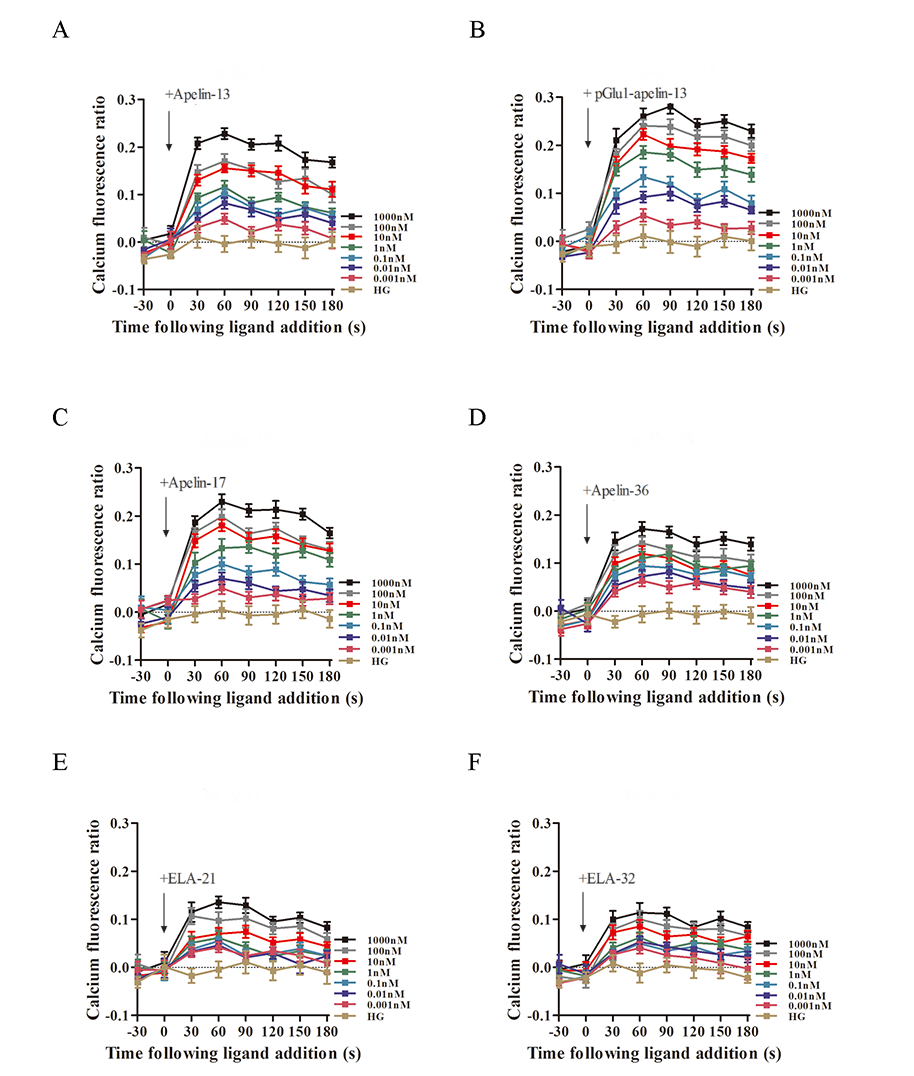


Supplementary Figure 3: The intracellular Calcium production induced by the six agonists. (A-F) Time-response curve of the agonists, in various concentrations, in HEK293 cells with stable expression of APJ.

P<0.05 was considered as statistically significant. Control group (HG, Cells were stimulated with high glucose). Data represent mean ± SEM from four independent experiments. Statistical analysis (Table S2)

4, Supplementary Figure 4


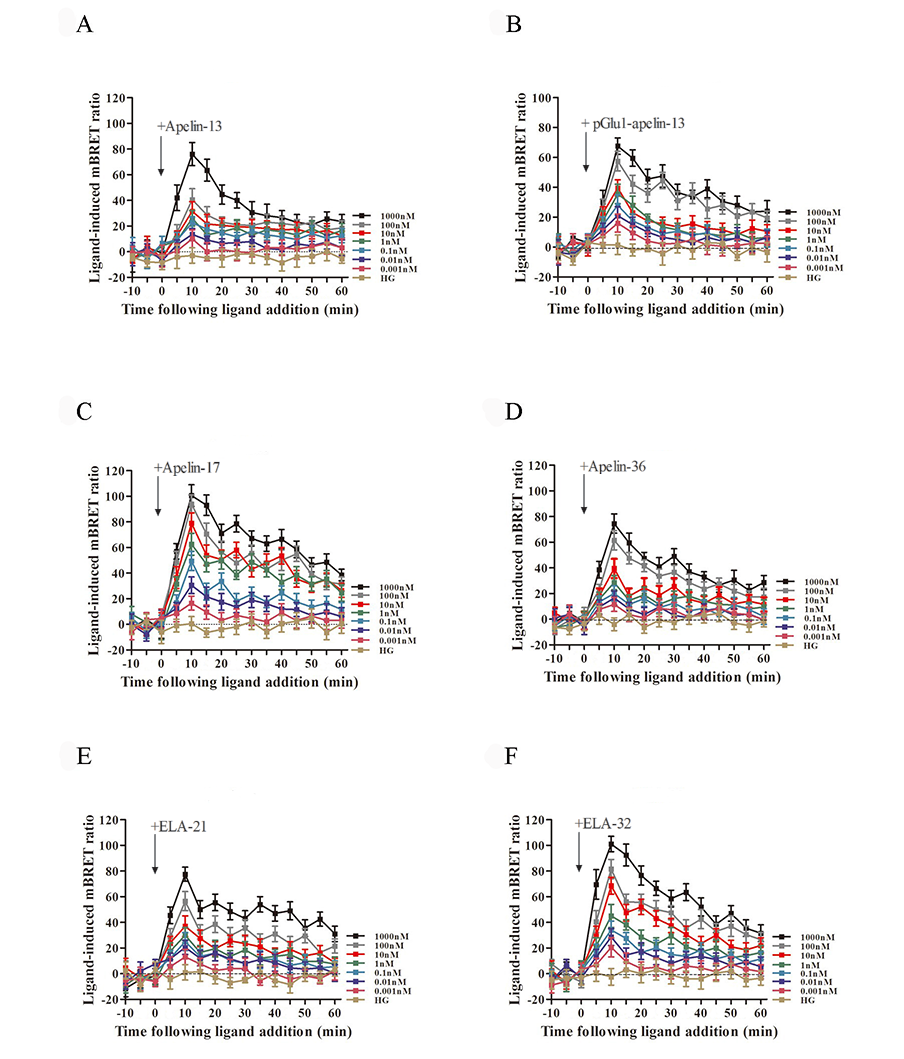


Supplementary Figure 4: The time-response curve (A-F for GRK2) of interactions between APJ and GRK2 induced by the six agonists of different concentrations, obtained from real-time dynamic BRET determination. P<0.05 was considered as statistically significant. Control group (HG, Cells were stimulated with high glucose). The data represent means ± SEM of four independent experiments. Statistical analysis (Table S3)
