# Supplementary figures and images for: The Effects of Apelin and Elabela Ligands on Apelin Receptor Distinct Signaling Profiles
Source: Front Pharmacol. 2021 Mar 4;12:630548. doi: 10.3389/fphar.2021.630548 (PMC7970304; doi:10.3389/fphar.2021.630548)

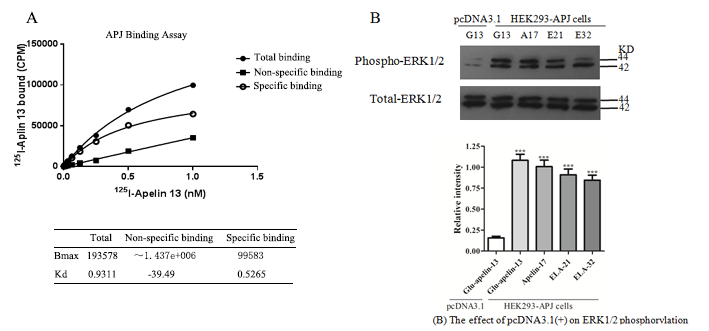

Supplement: Supplementary file 2 [file image1.tif]

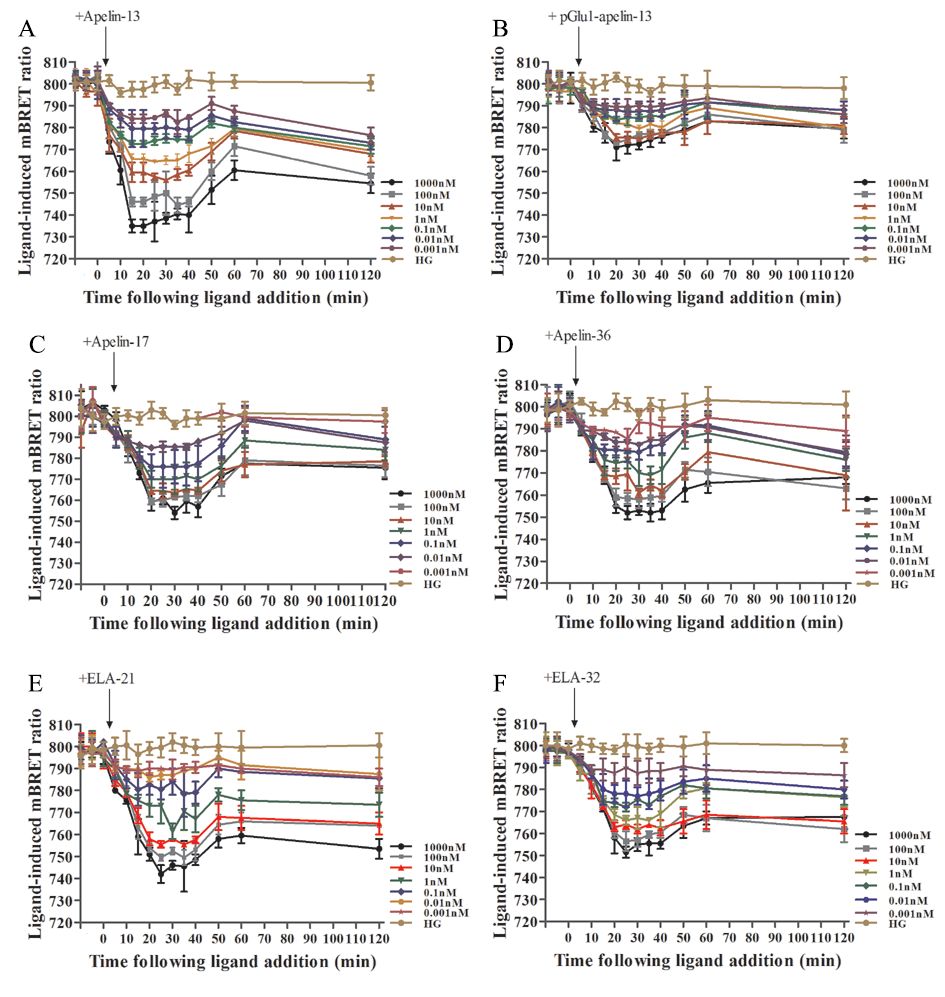

Supplement: Supplementary file 3 [file image2.tif]

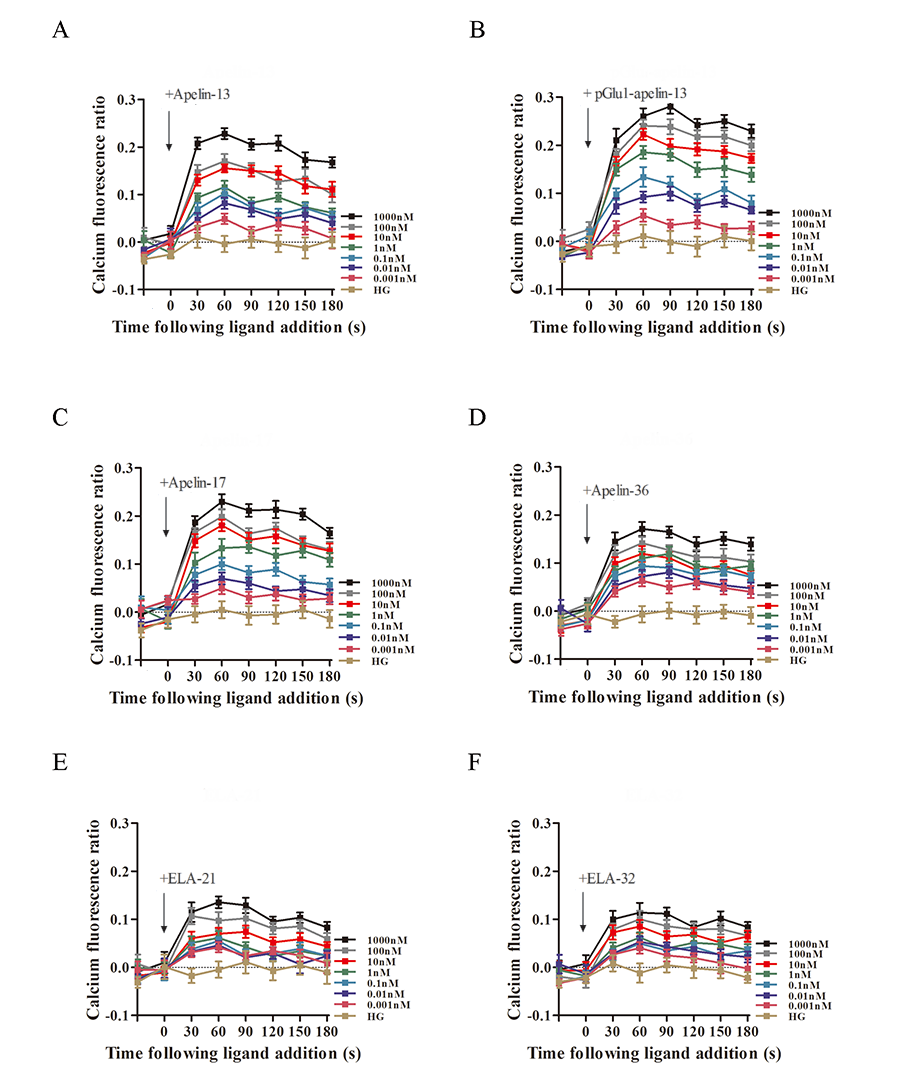

Supplement: Supplementary file 4 [file image3.tif]

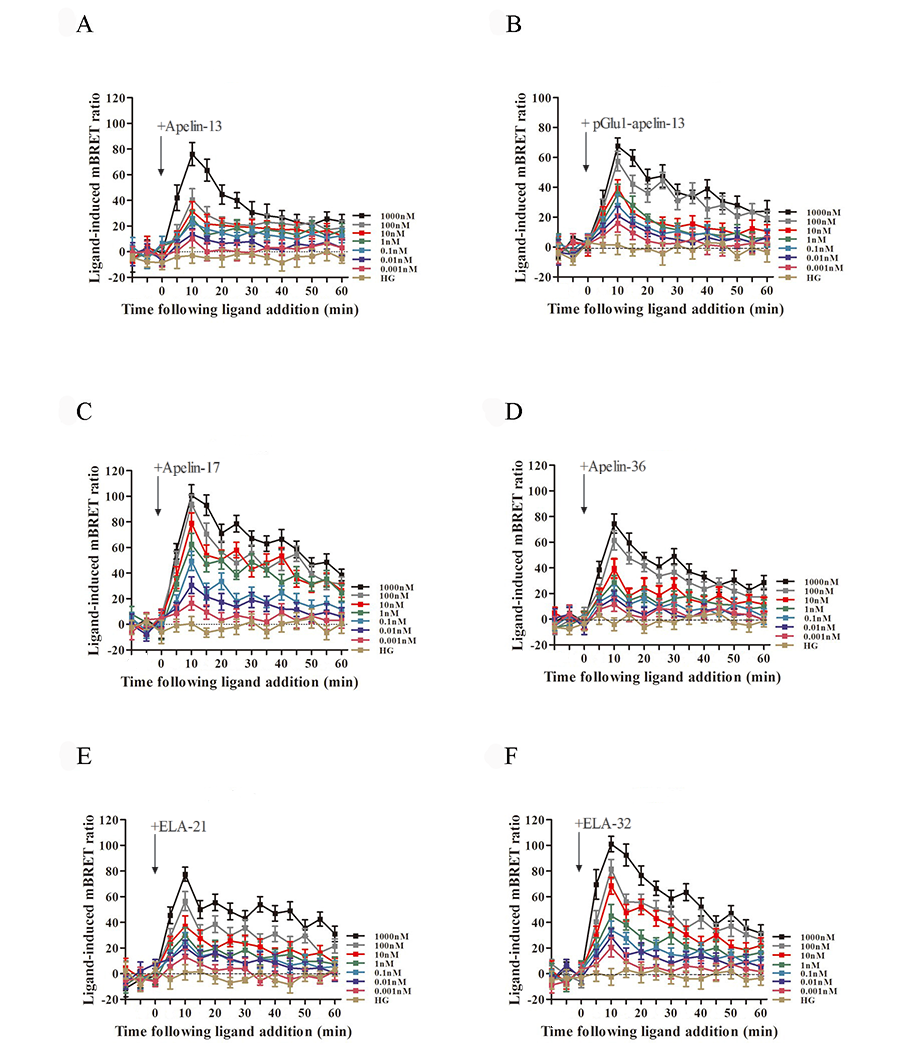

Supplement: Supplementary file 5 [file image4.tif]

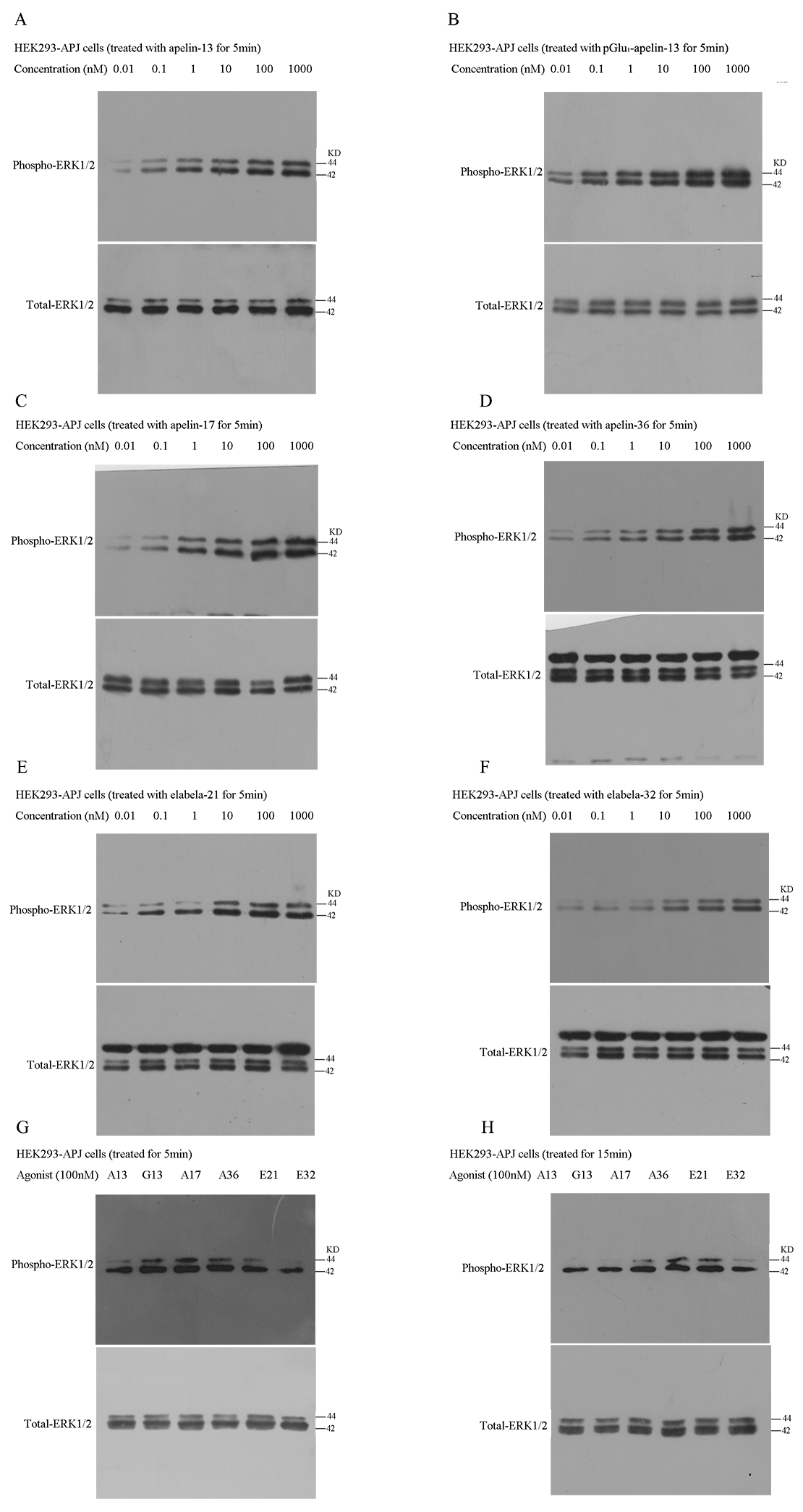

Supplement: Supplementary file 6 [file image5.tif]

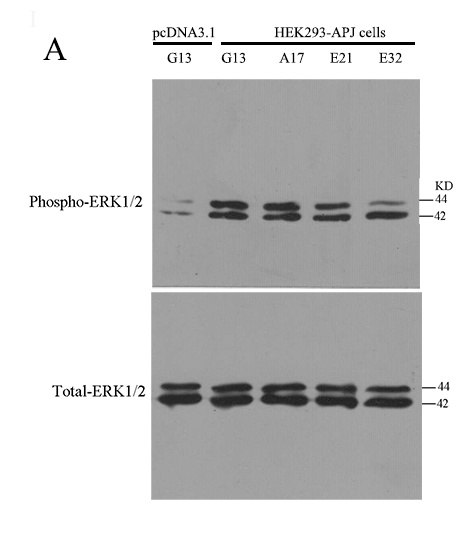

Supplement: Supplementary file 7 [file image6.tif]
